# *Reduced Anthocyanins in Petioles* codes for a GST anthocyanin transporter that is essential for the foliage and fruit coloration in strawberry

**DOI:** 10.1093/jxb/ery096

**Published:** 2018-03-10

**Authors:** Huifeng Luo, Cheng Dai, Yongping Li, Jia Feng, Zhongchi Liu, Chunying Kang

**Affiliations:** 1 Key Laboratory of Horticultural Plant Biology (Ministry of Education), College of Horticulture and Forestry Sciences, Huazhong Agricultural University, Wuhan, China; 2 College of Plant Science and Technology, Huazhong Agricultural University, Wuhan, China; 3 Department of Cell Biology and Molecular Genetics, University of Maryland, College Park, MD, USA

**Keywords:** Anthocyanin transporter, foliage coloration, *Fragaria vesca*, fruit coloration, glutathione S-transferase, mutant analysis, strawberry

## Abstract

The red color of the foliage and fruit in strawberry comes from anthocyanins stored in the vacuole; however, how this anthocyanin accumulation is regulated remains unclear. A *reduced anthocyanin in petioles* (*rap*) mutant was identified in an N-ethyl-N-nitrosourea (ENU) mutagenized population of YW5AF7, a white-fruited variety of the wild strawberry *Fragaria vesca*. The causative mutation was identified to be a premature stop codon in a *glutathione S-transferase* (*GST*) gene. In addition to the foliage coloration, *RAP* also mediates fruit pigmentation and acts downstream of the fruit-specific transcription factor *FvMYB10*. Among all eight *GST* genes in the same subfamily, *RAP* is most abundantly expressed in the ripening fruit. Expression analysis and transient expression assays demonstrated that RAP is the principal transporter of anthocyanins among the paralogs. Moreover, domain-swap experiments showed that both the N- and C-terminals of RAP are essential for the binding capability of anthocyanins. In addition, transient knock-down of *RAP* resulted in reduced fruit coloration in cultivated strawberry. Collectively, our results demonstrate that *RAP* encodes the principal GST transporter of anthocyanins in the strawberry foliage and fruit, and it could be modified to alter the fruit color in strawberry.

## Teaser

*Reduced Anthocyanins in Petioles* (*RAP*) encodes a glutathione S-transferase anthocyanin transporter that is essential for the foliage and fruit coloration in strawberry.

## Introduction

Cultivated strawberry (*Fragaria ananassa*) is one of the major fruit crops grown worldwide. The wild diploid *F. vesca* has emerged as a model plant for cultivated strawberry, as well as other Rosaceae fruit, because of the fact that it has a small stature, a short life cycle, its genome has been sequenced (~240 Mb; Shulaev *et al.*, 2011), and it is easy to transform (Oosumi *et al.*, 2006). In strawberry, the botanical fruits are the small and multiple achenes adhering to the receptacle surface, while the juicy flesh is derived from the enlarged stem tip that forms the receptacle. In recent years, extensive transcriptome datasets generated from *F. vesca* during flower and fruit development have provided a valuable resource for studying this unique receptacle and its development (Kang *et al.*, 2013; Hollender *et al.*, 2014; Li *et al.*, 2017). Our own research has frequently utilized three *F. vesca* accessions: the 7th inbred line of ‘Yellow Wonder’ (YW5AF7), ‘Hawaii 4’ (H4), and ‘Ruegen’. While all three accessions have red petioles, only Ruegen develops red fruit, including red receptacles and red achenes. YW5AF7 and H4, in contrast, bear white receptacles and white achenes. The loss of red color in YW5AF7 and H4 was recently shown to result from a natural mutation in the *FvMYB10* gene, which encodes a key transcription factor for anthocyanin biosynthesis (Hawkins *et al.*, 2016).

Anthocyanins are a group of water-soluble flavonoid compounds that perform diverse biological functions, such as attracting pollinators and seed dispersers (Schaefer *et al.*, 2004), conferring stress resistance, and prolonging fruit life span (Zhang *et al.*, 2013). Anthocyanins also give rise to the brilliant red strawberry fruit favored by consumers. Similar to other plant species, anthocyanins are synthesized in strawberry by a well-studied pathway with a series of enzymes using phenylalanine as the precursor (Pillet *et al.*, 2015). At the final step, a UDP-glucosyltransferase, such as UGT71K3 (Song *et al.*, 2016), catalyses the glucosylation of the anthocyanins to increase their stability. These anthocyanin biosynthetic enzymes contribute to fruit coloration in strawberry. Most of their coding genes are differentially expressed between the red- and white-fruited strawberry varieties (Zhang *et al.*, 2015; Hawkins *et al.*, 2017), and knock-down of their expression is able to alter the fruit color (Hoffmann *et al.*, 2006; Fischer *et al.*, 2014).

It has been demonstrated in several plant species that the genes coding for anthocyanin biosynthesis enzymes are subjected to transcriptional regulation by the well-studied ‘MBW’ complex. This consists of one MYB transcription factor, one bHLH transcription factor, and one WD-40 protein (Xu *et al.*, 2015). In strawberry, this complex has also been shown to be a master regulator of pigmentation. Overexpression of *FaMYB10* in *F. ananassa* or *FvMYB10* in *F. vesca* results in an accumulation of anthocyanins in the roots, leaves, and fruit (Lin-Wang *et al.*, 2010, 2014), whereas *Fa*/*FvMYB10*-RNAi transgenic lines produce white fruit (Lin-Wang *et al.*, 2014; Medina-Puche *et al.*, 2014). As referred to above, a natural SNP that causes an alteration in the amino acids from W (Trp) to S (Ser) at position 12 of FvMYB10 results in white fruit color in the H4 and YW accessions of *F. vesca* (Hawkins *et al.*, 2016). Hence, the genotype of H4 and YW is *myb10*, while the genotype of Ruegen is *MYB10*. In addition, FaMYB1, a different MYB transcription factor, is a transcriptional repressor of anthocyanin biosynthesis genes that acts in the last few steps of the flavonoid pathway (Aharoni *et al.*, 2001). More regulatory transcription factors have been identified by expression correlation analysis in strawberry (Pillet *et al.*, 2015), indicating that our knowledge of the gene network of this pathway is still incomplete.

Anthocyanins are synthesized at the endoplasmic reticulum, at the side that faces the cytoplasm, and are then transported into the vacuole for storage. Several types of mechanism are known to be responsible for anthocyanin transport in plants, including glutathione S-transferases (GSTs), multidrug and toxic extrusion (MATE), ATP-binding cassette (ABC) proteins, and possibly the allergen Fra a 1 (Goodman *et al.*, 2004; Marinova *et al.*, 2007; Gomez *et al.*, 2009; Muñoz *et al.*, 2010; Zhao *et al.*, 2011; Francisco *et al.*, 2013). Of particular interest in relation to the current study are the GSTs (EC 2.5.1.18), which are dimeric enzymes involved in cellular detoxification by conjugating glutathione (GSH) to a variety of electrophilic compounds (Dixon *et al.*, 2002). The functions of plant GSTs include detoxification of xenobiotics as well as responses to biotic and abiotic stresses (Loyall *et al.*, 2000; Agrawal *et al.*, 2002). GSTs comprise a large gene family in plant species, and are soluble and highly abundant in the cytosol (McGonigle *et al.*, 2000; Wagner *et al.*, 2002; Dixon *et al.*, 2009; Jain *et al.*, 2010). Plant GSTs can be divided into five subfamilies, namely phi, tau, theta, zeta, and lambda (Dixon *et al.*, 2002), with those involved in anthocyanin transport belonging to the plant-specific phi subfamily (Kitamura *et al.*, 2004). A typical GST protein contains a conserved GSH-binding site (G-site) located in the N-terminus domain and a C-terminus substrate-binding domain (H-site), with the two being in proximity of each other in a 3-D structure that forms the catalytic site (Dixon *et al.*, 2002).

Among the anthocyanin transporters, GSTs probably play the most important role as loss of their function causes a phenotype with a visible loss of pigment, such as *bz2* (*Bronze-2*) from maize, *an9* (*Anthocyanin 9*) from petunia, *fl3* (*Flavoniod 3*) from carnation, and *tt19* (*Transparent Testa 19*) from Arabidopsis (Marrs *et al.*, 1995; Alfenito *et al.*, 1998; Larsen *et al.*, 2003; Kitamura *et al.*, 2004; Sun *et al.*, 2012). Several studies have carefully examined the roles of *TT19* in anthocyanin accumulation in Arabidopsis and provided strong evidence that TT19 acts as a carrier protein for sequestration of anthocyanins from the cytosol into the vacuole (Kitamura *et al.*, 2004; Li *et al.*, 2011; Sun *et al.*, 2012). In fruit crops, GSTs have been associated with fruit or flower pigmentation, such as *LcGST4* from lychee, *MdGST* from apple, and *Riant* from peach (Cardoso *et al.*, 2012; Cheng *et al.*, 2015; El-Sharkawy *et al.*, 2015; Hu *et al.*, 2016). However, none of them has been studied using genetic approaches. In our current study, a *F. vesca* mutant produced as a result of an N-ethyl-N-nitrosourea (ENU) chemical mutagenesis of YW was found to accumulate very little anthocyanin in the leaf petioles and was thus named *reduced anthocyanins in petioles* (*rap*). Mapping by sequencing revealed that *RAP* encodes a GST transporter for anthocyanin. Using a combination of different approaches, this study demonstrates that RAP is the pivotal anthocyanin transporter in the foliage and fruit of strawberry, thus providing a promising candidate gene for improving or manipulating fruit and foliage color in cultivated strawberry.

## Materials and methods

### Plant material and ENU mutant screening

The 7th generation inbred lines of three *F. vesca* accessions, namely Yellow Wonder 5AF7 (YW5AF7, white-fruited), Ruegen (Ru F7-4, red-fruited), and Hawaii 4 (PI551572, National Clonal Germplasm Repository, USA, white-fruited), were used as wild-types in this study (Slovin *et al.*, 2009; Hawkins *et al.*, 2016). The plants were cultivated in a growth room under a light intensity of 100 μmol m^−2^ s^−1^ with a 16/8 h light/dark photoperiod at 22 °C. For ENU mutagenesis, the seeds of YW5AF7 were first soaked in water for 1 d at 4 °C, then treated with 0.4% ENU (N3385, Sigma-Aldrich) for 8 h at room temperature with gentle shaking, and finally rinsed thoroughly 10 times with water (Caruana *et al.*, 2018). After storage at 4 °C for 2 weeks, the seeds were propagated in a greenhouse. Mutants were screened in the M2 generation.

### Identification and isolation of *RAP* via mapping-by-sequencing

The *rap* mutant in the M3 generation was backcrossed into the parent YW5AF7 to generate an F_2_ population. In this population, equal amounts of young leaves were pooled from 27 wild-type plants and 18 mutant plants. DNA extraction for the two groups was performed using a CTAB method (Porebski *et al.*, 1997; Oosumi *et al.*, 2006). A total of 6G paired-end reads at 150 bp were generated for each of the two groups using the Illumina HiSeq X Ten platform (Biomarker Technologies, Beijing). The reads were aligned to the v2.0 genome of *F. vesca* using Bowtie2 (http://sourceforge.net/projects/bowtie-bio/files/bowtie2/2.2.6/) (Tennessen *et al.*, 2014). The SNPs were called by SAMtools (https://sourceforge.net/projects/samtools/files/samtools/). The following criteria were applied to filter the SNPs: 100% presence in the mutant, <50% in the wild-type, and absent in the wild-type parent YW5AF7. In addition, the candidate SNPs had to be located in the exon and cause non-synonymous or nonsense mutations in the protein product. After passing these filtering criteria, the resulting candidate SNPs were further confirmed by PCR-amplification and Sanger sequencing in each individual *rap* mutant in the F_2_ population.

### Phylogenetic analysis

The protein sequences were downloaded from PLAZA (Proost *et al.*, 2015) or NCBI (https://www.ncbi.nlm.nih.gov/) according to the accession numbers (see Results). Protein sequences of the six mis-annotated GST-encoding genes in *F. vesca* (*RAP*, *RAP-L1*, *RAP-L2*, *RAP-L4*, *RAP-L5*, and *RAP-L7*) were obtained from the new *F. vesca* annotation v2.0.a2 (Supplementary Fig. S3 at *JXB* online) (Li *et al.*, 2018). The sequence alignment was performed using Clustal Omega (http://www.ebi.ac.uk/Tools/msa/clustalo). An unrooted phylogenetic tree was constructed using MEGA 7 (http://www.megasoftware.net/) with the neighbor-joining statistical method and bootstrap analysis (1000 replicates).

### Plasmid construction

The primers used for plasmid construction are listed in Supplementary Table S1. Genomic DNA or cDNA obtained from fruit or young leaves of Ruegen were used for sequence amplification. For complementation rescue, the genomic sequence of *RAP* from 1114 bp upstream of the translation start site to 1182 bp downstream of the stop codon (3317 bp in total) was amplified, sub-cloned into pDONR221, and then inserted into the binary vector pMDC99. For overexpression, *RAP-RFP*, *MYB10*, *RAP-L1-7*, and the two chimeric genes of *RAP* and *RAP-L1* (Supplementary Fig. S4B) were cloned into either pDONR221 or pENTR1A and inserted into the binary vector pK7WG2D. For RNAi, the entire coding sequence of *RAP* from 1 to 512 bp was inserted into the binary vector pK7GWIWG2D. To examine the subcellular localization, coding sequences of *RAP* and *RAP-L1-5* were cloned into the EcoRI- and NdeI-digested binary vector pRI101 to fuse with *GFP* (green fluorescent protein) using the Gibson cloning method. These constructs were transformed into *Agrobacterium tumefaciens* strain GV3101 for plant transformation.

### Stable transformation in Arabidopsis

Arabidopsis transformation was carried out by the floral-dip method (Clough and Bent, 1998). T1 transgenic seeds were screened on half-strength Murashige and Skoog (MS) medium (M5524, Sigma-Aldrich) with 100 mg l^–1^ kanamycin.

### Transient gene expression in strawberry fruit

Transient expression assays in strawberry fruit were performed as described by Hoffmann *et al.* (2006). Briefly, a single *Agrobacterium* colony was selected and grown in 2 ml of liquid LB medium until OD_600_ reached about 0.8–1.0. The culture was then spun down and resuspended in buffer (1×MS, 2% sucrose) to reach an OD_600_ of exactly 0.8. Fruit at the white stage were injected using a 10-ml syringe. The color phenotype was examined 1 week after the injection, and at least 10 fruit were used for each construct.

### Transient gene expression in tobacco leaves and microscopy

Transient expression in tobacco leaves was performed as described by Sparkes *et al.* (2006). The fluorescence in transformed cells was observed using a confocal microscope (Leica, SP8). GFP was excited at 488 nm and captured at 500–530 nm. Red (RFP) and orange fluorescent proteins (OFP) were excited at 543 nm and captured at 560–630 nm. Chlorophyll autofluorescence was captured at 650–750nm.

### Quantitative RT-PCR

Total RNA was extracted using a Plant Total RNA Isolation Kit (Sangon Biotech, Shanghai, China, No. SK8631) following the manufacturer’s instructions. Approximately 1 μg of total RNA was used for cDNA synthesis using a PrimeScript^TM^ RT reagent kit (TaKaRa, Shiga, Japan, Cat# RR047A). For qPCR, a total volume of 10 μl reaction mixture was used containing 5 μl of 2×SYBR Green master mix (Cat# 172–5124, BioRad), 1 μl of 5× diluted cDNA, 0.25 μl of each primer, and 3.5 μl ddH_2_O (Supplementary Table S2). Amplification was performed using a QuantStudio 7 Flex system (Applied Biosystems, USA). The amplification program consisted of one cycle of 50 °C for 2 min and 95 °C for 10 min, followed by 50 cycles of 95 °C for 15 s, 60 °C for 20 s, and 72 °C for 20 s. The fluorescent product was detected at the third step of each cycle. The expression level of each gene was calculated using the 2^−∆∆*C*T^ method (Livak and Schmittgen, 2001). All analyses were repeated three times using biological replicates.

### Measurement of total anthocyanins

Approximately 0.5 g fresh tissue was ground in liquid nitrogen, added to 5 ml of extraction solution (methanol: H_2_O: formic acid: trifluoroacetic acid, 70:27:2:1), and kept at 4 °C for 12 h in the dark. The supernatant was transferred to a new tube by filtration. The absorbance was measured at 530 and 657 nm by Hoefer Vision (SP-2001). The anthocyanin content was calculated using the following formula: QAnthocyanins = [A_530_−(0.25×A_657_)]/M, where QAnthocyanins is the amount of anthocyanins, A_530_ and A_657_ are the absorbance at the indicated wavelengths, and M is the fresh weight of the plant material used for extraction (Zhang *et al.*, 2009). All samples were measured as triplicates in three independent biological replicates.

### HPLC analysis of anthocyanins in strawberry petioles and fruit

Approximately 0.5 g fresh tissue was ground in liquid nitrogen, added to 2.5 ml of extraction solution (methanol: H_2_O: hydrochloric acid, 80:20:0.1), and kept at 4 °C for 12 h in the dark. The mixture was centrifuged at 9000 *g* for 20 min. The supernatant was filtered through a 0.45-μm millipore membrane. HPLC analysis was performed using a Daojing LC-20AT system. Separation was performed using a Develosil-ODS C18, 5-μm, 4.6 × 250-mm column. The mobile phase was 0.1% formic acid in water (solvent A) and methanol (solvent B) at a flow rate of 0.6 ml min^–1^. The linear gradient of phase B was as follows: 0–10 min, 10–25%; 10–15 min, 25–30%; 15–50 min, 30–50%; 50–60 min, 50–60%; 60–68 min, 60–10%; 68–70 min, 10%. The UV-visible light detector wavelength was set at 510 nm for detecting anthocyanins. Cyanidin (Cy) 3-gluc (Aladdin, 27661-36-5) was used as the authentic standard.

### Statistical analyses

For the segregation ratio test of the F_2_ population, the χ^2^ value was calculated manually. Statistical analyses were performed using SPSS (IBM SPSS Statistics v22.0).

## Results

### Identification of a reduced-color mutant *rap* in *F. vesca* and *RAP* gene isolation

**Fig. 1.**
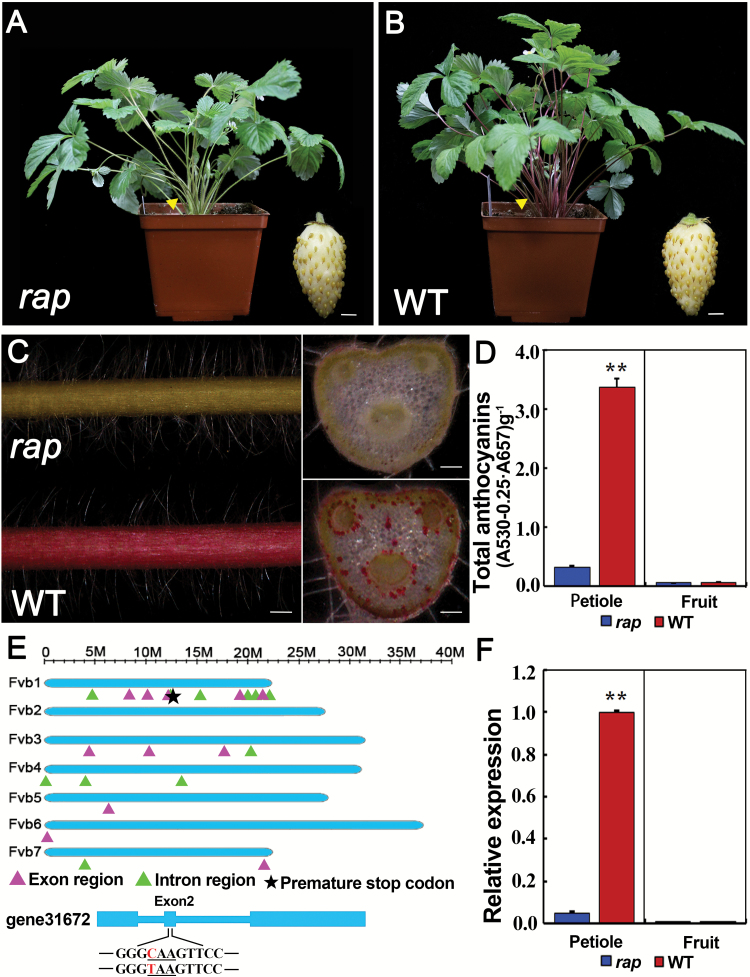
Phenotypes and gene isolation of the *rap* mutant. Images of the plant and mature fruit (inset) of (A) the *rap* mutant and (B) the wild-type (WT) YW5AF7. (C) Images showing the epidermis and cross-sections of a petiole of *rap* and YW5AF7 (WT). (D) Total anthocyanin contents in the petioles of mature leaves and in mature fruit of *rap* and YW5AF7 (WT). **, *P*<0.01, Student’s *t*-test. (E) Diagram showing the locations of high-quality exonic and intronic SNPs. The chromosome length is indicated at the top. SNPs located in exons or introns are indicated. The star indicates the SNP in gene31672 that causes a premature stop codon. In the model of gene31672, the three underlined nucleotides indicate the codon affected, and the SNP (C to T) concerned is in red. (F) Expression levels of *RAP* in the petioles of mature leaves and in mature fruit of *rap* and YW5AF7 (WT) as analysed by qRT-PCR. *Gene11892* was used as the internal control. Data are means (±SD) obtained from three technical replicates. **, *P*<0.01, Student’s *t*-test. The experiment was repeated three times with similar results. Scale bars: (A, B) 3 mm; (C) 1 mm (left), and 0.3 mm (right).

To identify genes essential for the regulation of fruit development in *F. vesca*, we mutagenized YW5AF7, the 7th inbred line of the strawberry variety Yellow Wonder, using the chemical mutagen ENU (Justice, 2000). In the M2 generation, a mutant with green petioles and leaves was identified and named as *rap* (*reduced anthocyanins in petioles*) (Fig. 1A, B). Closer observation showed that the pigmentation of epidermal cells was greatly reduced in the *rap* mutant, in contrast to the bright-red color in leaf petioles of the wild-type (YW5AF7). Similarly, cross-sections of leaf petioles showed the same change in some cortex cells, especially around the vasculature (Fig. 1C). Consistent with the reduced-pigment phenotype, the total anthocyanin content was remarkably reduced in the leaf petioles of *rap* (*P*<0.01, Student’s *t*-test), and very low in the fruit of both *rap* and the YW5AF7 wild-type control (Fig. 1D).

To examine whether a known gene in the anthocyanin pathway was mutated, the expression levels of catalytic enzyme genes (including *CHS*, *FSH*, *ANS*) and regulatory genes (*MYB10*, *MYB1*, *bHLH33*, and *bHLH3*) were compared between *rap* and YW5AF7 in the leaf petioles by qRT-PCR (Supplementary Fig. S1A). A majority of these genes were either similarly or more highly expressed in *rap*; however, the expression of *LAR* and *MYB10* was significantly reduced (Supplementary Fig. S1B). The coding sequences of *MYB10* and *LAR* were subsequently examined, but neither was found to harbor any mutation. These results indicated that none of the examined genes had the causative mutation for *rap*.

To identify the causative mutation, the *rap* mutant was backcrossed with YW5AF7 to generate the F_2_ mapping population. In this population, the ratio of mutant to wild-type plants was 48:137, which is close to 1:3 (χ^2^=0.045; χ^2^_0.05_=3.84). Young leaves from the F_2_ mutant group (18 plants) and the F_2_ wild-type group (27 plants) were respectively pooled for whole-genome resequencing. Totals of 43.3 million and 48.5 million paired-end reads at 150 bp were obtained for the mutant and wild-type groups, respectively. We found that 93.7% of the mutant reads and 86.8% of the wild-type reads were aligned to the updated *F. vesca* genome (Fvb) (Tennessen *et al.*, 2014). SNP calling and filtering (see Methods) identified a total of 64 high-quality SNPs, of which 41 were in the intergenic regions, 11 in introns, three in UTRs, and nine in coding sequences. The locations of the exonic and intronic SNPs in each chromosome are indicated in Fig. 1E. It was notable that more SNPs were located in Chromosome 1 than in other chromosomes. Among the SNPs in the coding region, eight caused either a synonymous or a missense mutation, whilst only one SNP (C to T) in the 2nd exon of *gene31672* resulted in a premature stop codon (Fig. 1E, indicated by a star in Chromosome 1). Out of the 31 mapped reads from the genome resequencing data of the mutant group, this position had 31 Ts, i.e. a SNP index of 100%, while the wild-type group had 5 Ts out of the 24 mapped reads, a SNP index of 20.8% (Supplementary Fig. S2). We then examined this SNP individually in 50 F_2_*rap* mutants by PCR-amplification and Sanger sequencing, and found that all 50 were homozygous for the mutation. Moreover, the expression level of *RAP* was greatly reduced in the petioles of *rap* (Fig. 1F), indicating nonsense-mediated decay for the *rap* mutant mRNA. Taking the results together, *gene31672* was identified as the primary candidate for *RAP*.

### Characterization of *RAP* and its paralogs in *F. vesca*

**Fig. 2.**
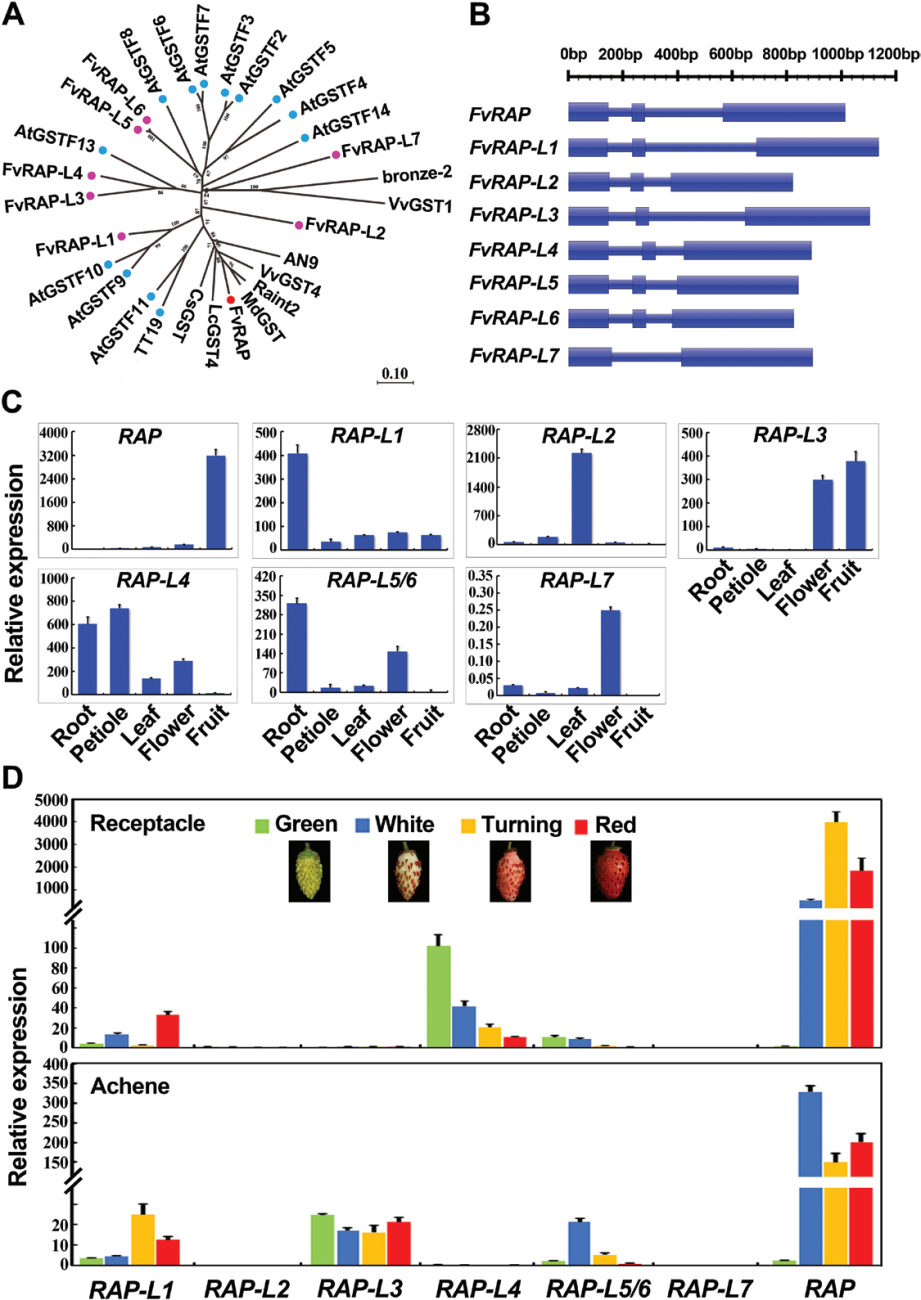
Phylogenetic analyses and expression patterns of *RAP* and its paralogs. (A) Phylogenetic tree of *RAP* and its homologs. A neighbor-joining tree was constructed based on protein sequences of RAP and its homologs from *F. vesca*, Arabidopsis, and several other species. Gene IDs are shown for genes from *F. vesca* and *Arabidopsis*, while the accession numbers in NCBI are shown for others: Riant2 (KT312848), MdGST (AEN84869), AN9 (Y07721), bronze-2 (AAV64226), VvGST4 (AAX81329), VvGST1 (AAN85826), LcGST4 (KT946768), CsGST (ABA42223), FvRAP (gene31672), FvRAP-L1 (gene28763), FvRAP-L2 (gene08595), FvRAP-L3 (gene22014), FvRAP-L4 (gene10549), FvRAP-L5 (gene10550), FvRAP-L6 (gene10551), FvRAP-L7 (gene10552), AtGSTF2 (At4G02520), AtGSTF3 (At2G02930), AtGSTF4 (At1G02950), AtGSTF5 (At1G02940), AtGSTF6 (At1G02930), AtGSTF7 (At1G02920), AtGSTF8 (At2G47730), AtGSTF9 (At2G30860), AtGSTF10 (At2G30870), AtGSTF11 (At3G03190), AtGSTF12/TT19 (At5G17220), AtGSTF13 (At3G62760), and AtGSTF14 (At1G49860). The numbers indicate the bootstrap values calculated from 1000 replicate analyses. (B) Gene models of *RAP* and its paralogs. Thick bars indicate exons, and thin bars indicate introns. The sequence length is shown at the top. (C) Expression patterns of *RAP* and its paralogs in the tissues of red-fruited Ruegen as analysed by qRT-PCR. (D) Expression patterns of *RAP* and its paralogs in fruit receptacles and achenes of Ruegen at four developmental stages, as analysed by qRT-PCR. *Gene11892* was used as the internal control in (C) and (D). Data are means (±SD) obtained from three technical replicates. The experiment was repeated for three times with similar results.

Sequence analysis indicated that *RAP* encodes a glutathione S-transferase (GST). A BLAST search identified seven other GSTs (Supplementary Fig. S3) in the *F. vesca* genome that share a high level of similarity to *RAP*, spanning the entire coding sequence (Supplementary Fig. S4A). They were named as *RAP-L1* to *RAP-L7* (*RAP-Like* 1–7). As shown in the phylogenetic tree (Fig. 2A), the eight *F. vesca* GSTs were closely related to the 13 Arabidopsis GSTs in the phi subfamily (Dixon and Edwards, 2010). The closest homologs of RAP were Riant2 from peach and MdGST from apple, two species in the Rosacea family. *RAP-L*5 to *RAP-L7* were next to each other in Chromosome 2 (Fvb2), perhaps due to gene duplications. The gene models of the *RAP* paralogs were quite similar: seven of them possessed three exons, with the exception of *RAP-L7* that had two exons (Fig. 2B).

The expression patterns of the *RAP* and *RAP-Like* genes were examined by qRT-PCR using gene-specific primers (Supplementary Table S2) in the roots, petioles of mature leaves, unfolded leaves, flowers (pooled from entire flower buds at different developmental stages), and mature fruit of the red-fruited Ruegen. The genes exhibited a great diversity in expression patterns (Fig. 2C). *RAP* was predominantly expressed in fruit, *RAP-L1* was more abundant in roots, *RAP-L2* was leaf specific, and *RAP-L3* was expressed in flowers and fruit. The expression level of *RAP-L4* was higher in roots and petioles. *RAP-L5/6*, the combined expression of *RAP-L5* and *RAP-L6* due to their high sequence similarity, was expressed in roots and flowers. *RAP-L7* was expressed at low levels compared to the other *RAP-Like* genes, but was found to be more abundant in flowers.

To explore their possible contributions to fruit coloration, the expression trends of *RAP* and its paralogs in fruit receptacles and achenes were investigated during fruit ripening. Four developmental stages (green, white, turning, and red) were examined. In the fruit receptacles, *RAP-L4* was most abundant at the green stage. In the subsequent stages, coinciding with the coloration period, only *RAP* increased in expression, and this increase was large (Fig. 2D). In the achenes, *RAP* was also the most abundantly expressed gene during the white, turning, and red stages, while the other *RAP-like* genes remained at low expression levels at all four stages (Fig. 2D). These results suggested that *RAP* probably plays more important roles than its homologs in the coloration of fruit receptacles and achenes during *F. vesca* fruit development.

### *RAP* is the ortholog of *TT19* in Arabidopsis

**Fig. 3.**
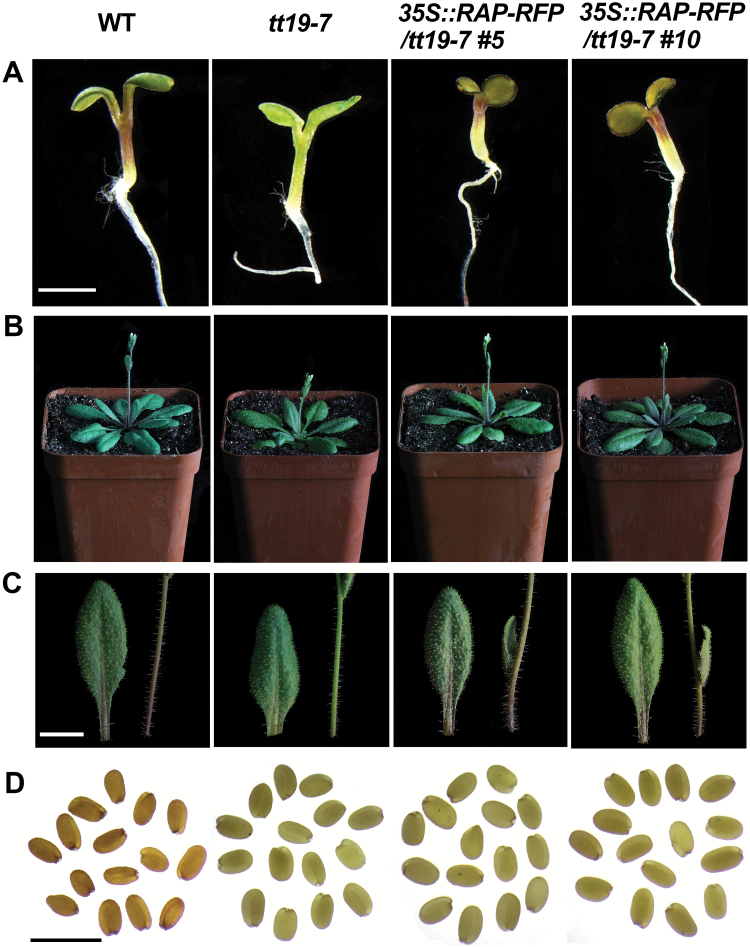
Phenotypes of the *35S::RAP-RFP* transgenic lines in Arabidopsis *tt19-7*. (A) Images of 7-d-old seedlings grown on MS medium supplemented with 5% sucrose. (B) Images of the adult plants at bolting. (C) Images of leaves and stems from the adult plants in (B). (D) Images of fresh seeds. Images for the wild-type (WT, Col), *tt19-7*, and two transgenic lines of *35S::RAP-RFP* (L5, L10) in the *tt19-7* background are shown. Scale bars: (A–C) 5 mm; (D) 1 mm.

TT19 is the homolog of RAP in Arabidopsis, and has been demonstrated to be an anthocyanin transporter (Sun *et al.*, 2012). To test the role of *RAP* in anthocyanin transport, *35S::RAP-RFP* was transformed into the Arabidopsis mutant *tt19-7*. A total of 14 independent transgenic lines were obtained with similar phenotypes, and two of them (Line5 and Line10) with high expression levels of *RAP* validated by qRT-PCR (Supplementary Fig. S5) were chosen for careful characterization. Seeds of wild-type, *tt19-7*, and the two lines of *35S::RAP-RFP*; *tt19-7* were germinated on MS medium supplemented with 5% sucrose. At 7 d post germination, hypocotyls of *tt19-7* were green, while the *35S::RAP-RFP*; *tt19-7* seedlings had red hypocotyls identical to the wild-type (Fig. 3A). Consistently, both stems and leaves of the transgenic plants accumulated more anthocyanins than *tt19-7* when grown in the growth room without any treatment (Fig. 3B, C). Since *RAP* can complement *tt19*-7, this suggested that RAP is an anthocyanin transporter. However, the brown color of seed coats was not rescued in the *35S::RAP-RFP* transgenic lines (Fig. 3D), suggesting that RAP may have distinct functions from TT19 during seed-coat pigmentation.

### *RAP* is essential for fruit coloration and acts downstream of *FvMYB10* in *F. vesca*

**Fig. 4.**
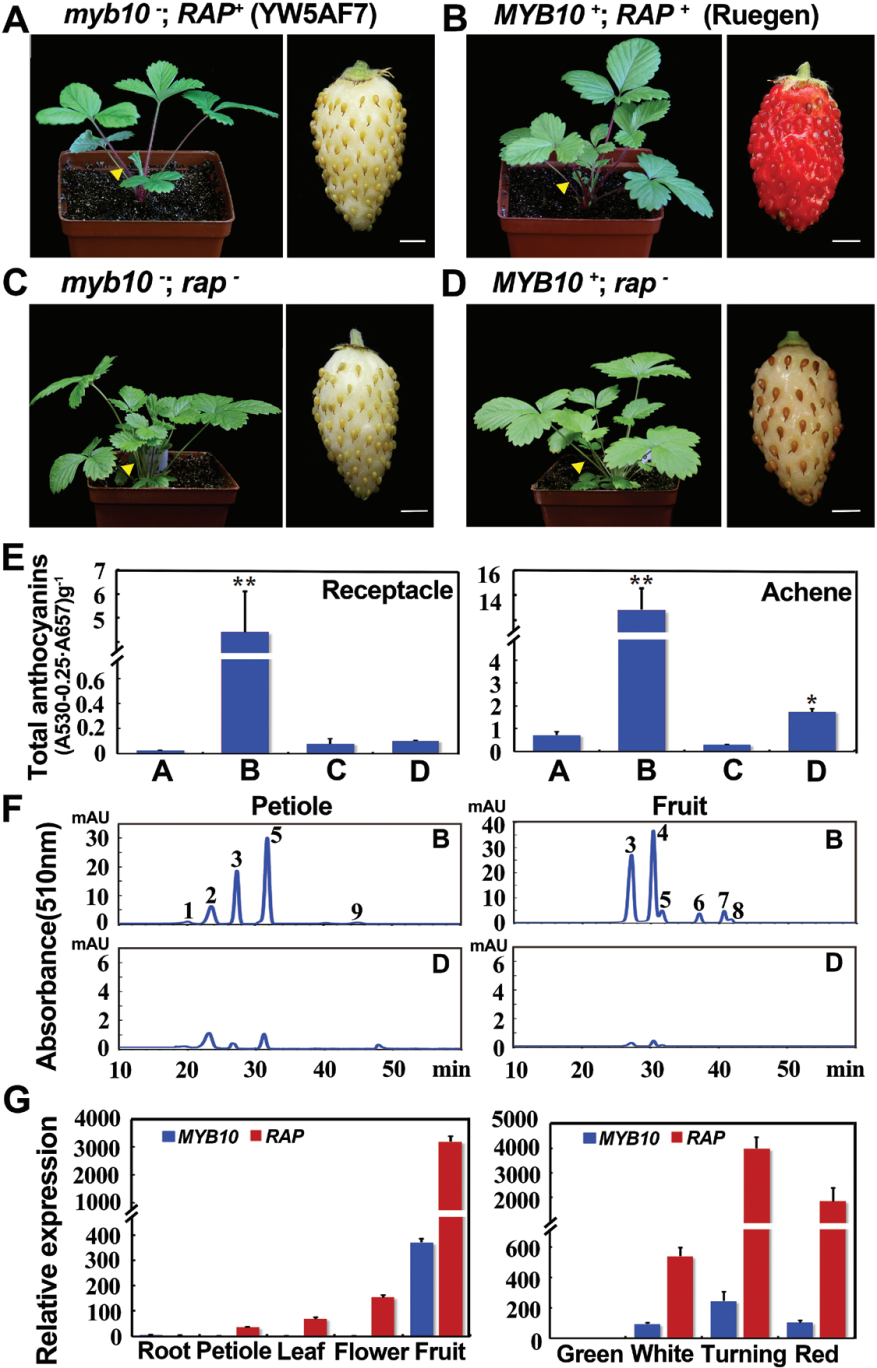
The function of *RAP* in fruit coloration. Images showing the plant and mature fruit of (A) YW5AF7, (B) Ruegen, (C) *rap*, and (D) *MYB10*^*+*^*rap*^−^. The genotypes are indicated above each set of images. Yellow arrowheads point to petioles. (E) Total anthocyanin contents in fruit receptacles and achenes collected from the plants shown in (A–D). The values of B–D are compared to A: *, *P*<0.05, **, *P*<0.01, Student’s *t*-test. (F) HPLC chromatograms of anthocyanins in petioles of mature leaves and mature fruit of Ruegen (as shown in B) and *MYB10*^*+*^*rap*^−^ (as shown in D). The *y*-axis shows the absorbance at 510 nm. Peak 1, cyanidin-3,5-diglucoside; peak 2, peonidin-3,5-diglucoside; peak 3, cyanidin-3-glucoside; peak 4, pelargonidin-3-glucoside; peak 5, peonidin-3-glucoside; peak 6, cyanidin-3-malonylglucoside; peak 7, pelargonidin-3-acetylhexoside; peak 8, peonidin-3-malonylglucoside; peak 9, unknown. (G) Co-expression of *RAP* and *MYB10* in different tissues (left) and in fruit receptacles at four developmental stages of Ruegen (right) as analysed by qRT-PCR. *Gene11892* was used as the internal control. Data are means (±SD) obtained from three technical replicates. The experiment was repeated three times with similar results. Scale bars in (A–D) are 3 mm.

The *rap* mutant was isolated from the white-fruited and red-petiole parent YW5AF7 (genotype: *myb10*^−^) and hence *rap* is actually a *myb10*^−^*rap*^−^ double-mutant (Fig. 4A, C). This makes it impossible to determine the role of *RAP* in fruit coloration. To examine whether *RAP* is involved in fruit pigmentation, we sought to isolate the single *rap* mutant. *rap* was crossed with the red-fruited Ruegen (genotype: *MYB10*^*+*^*RAP*^*+*^) (Fig. 4B). In the F_2_ population, the plants with green petioles, like those of *rap* (Fig. 4C), were selected. Among these green-petiole plants, the genotype of *myb10* was identified via sequencing of the SNP in *FvMYB10/gene31413* (Hawkins *et al.*, 2016). Most plants were still *myb10*^−^*rap*^−^ due to the linkage of these two genes on Chromosome 1, and only one *MYB10*^*+*^*rap*^−^ plant was identified out of 66 green-petiole F_2_ plants. The isolation of the *rap* single mutant (*MYB10*^*+*^*rap*^−^) enabled us to characterize the effects of *RAP* without the interference of the background *myb10* mutation.

Mature fruit of *MYB10*^*+*^*rap*^−^ had white receptacles that were identical to YW5AF7, while the mature achenes were light pink, which was much less coloration than we observed in the achenes of Ruegen (Fig. 4B, D). This suggested that *rap* alone can affect fruit coloration both in the receptacle and the achenes. To characterize the phenotypes quantitatively, we measured the total anthocyanin contents of fruit receptacles and achenes of the *MYB10*^*+*^*rap*^−^ plant. The content in the receptacles of *MYB10*^*+*^*rap*^−^ was as low as that in *myb10*^−^*rap*^−^, but the content in the achenes of *MYB10*^*+*^*rap*^−^ was slightly higher than that of *myb10*^−^*rap*^−^ (*P*<0.05, Student’s *t*-test). Nevertheless, the anthocyanin contents in the receptacle and achenes of *MYB10*^*+*^*rap*^−^ were significantly reduced when compared with Ruegen (*MYB10*^*+*^*RAP*^*+*^) (Fig. 4E). Previous studies of Ruegen and YW5AF7 have shown that anthocyanins in petioles and fruit contain a total of nine prominent compounds, eight of which have been identified (Xu *et al.*, 2014). Using similar methods, we were able to observe and identify the same number of HPLC peaks in Ruegen and found that all the peaks were significantly reduced in *MYB10*^*+*^*rap*^−^ (Fig. 4F).

This genetic experiment indicated that both *MYB10* and *RAP* are required for fruit coloration. To determine the relationship between these two genes, we examined the expression of *RAP* and *MYB10* using qRT-PCR and found that *RAP* expression nicely correlated with *MYB10* expression in different tissues/organs and at different stages of fruit development (Fig. 4G). Moreover, proper expression of *RAP* relied on the wild-type *MYB10*, as shown in the three *F. vesca* varieties. Specifically, YW5AF7 and H4 produced white fruit with nearly no anthocyanin, while Ruegen had red fruit with high anthocyanin content (Supplementary Fig. S6A). The qRT-PCR results showed that *RAP* had low expression levels in YW5AF7 and H4, the two white-fruited varieties containing the *myb10* mutation, and was highly expressed in Ruegen, the red-fruited variety with the wild-type *MYB10* (Supplementary Fig. S6B), suggesting that *MYB10* probably regulates *RAP* expression in fruit either directly or indirectly. Of note, the expression level of *RAP* was comparable in the petioles of YW5AF7, H4, and Ruegen (Supplementary Fig. S6B), suggesting the existence of a different *MYB* that may act in the petiole for *RAP* expression.

### Expression of *RAP* rescued fruit pigmentation of *rap* in a transient assay

**Fig. 5.**
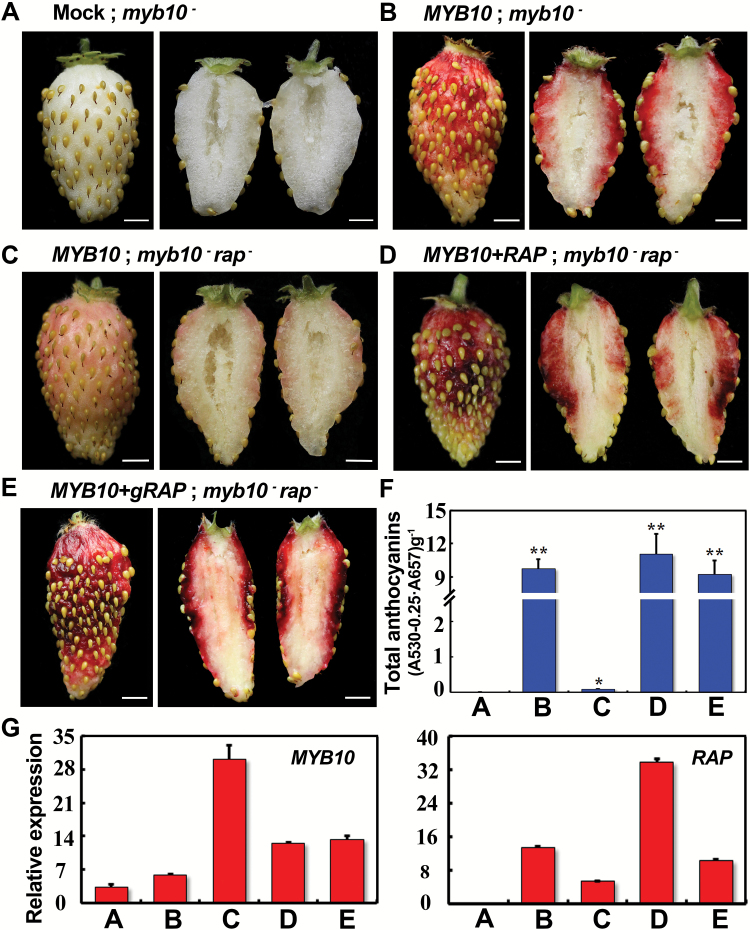
Rescue of the fruit coloration in *rap* in a transient assay. (A) A fruit of YW5AF7 (genotype: *myb10*^−^) infiltrated with buffer. (B) A fruit of YW5AF7 overexpressing *MYB10* by agro-infiltration. (C) A fruit of *rap* overexpressing *MYB10* by agro-infiltration. (D) A fruit of *rap* overexpressing both *MYB10* and *RAP* by agro-infiltration. (E) A fruit of *rap* expressing both *gRAP::RAP* and *35S::MYB10* by agro-infiltration. In each case, one representative example is shown from at least 10 infiltrated fruit. (F) Total anthocyanin contents in the fruit shown in (A–E). The values of B–E are compared to A: *, *P*<0.05, **, *P*<0.01, Student’s *t*-test. (G) Expression levels of *MYB10* and *RAP* in the fruit shown in (A–E) as analysed by qRT-PCR. *Gene11892* was used as the internal control. Data are means (±SD) obtained from three technical replicates. The experiment was repeated three times with similar results. Scale bars in (A–E) are 3 mm.

It has previously been shown that fruit pigmentation in YW5AF7 (genotype: *myb10*) can be rescued by overexpressing a functional *FvMYB10* via agro-infiltration (Hawkins *et al.*, 2016), providing a quick assay to test gene function in anthocyanin accumulation. Using this assay, we found that 35S::*FvMYB10* indeed reliably restored fruit coloration (Fig. 5A, B). Accordingly, *35S::FvMYB10* was infiltrated into the *rap* fruit, but they only exhibited a light-pink color (Fig. 5C), indicating that a functional *RAP* was necessary to restore the coloration. When *35S::RAP-RFP* and *35S::MYB10* were simultaneously infiltrated into the *rap* fruit, they turned bright red (Fig. 5D). When the genomic fragment of *RAP* (*gRAP::RAP*) was transiently transformed into the *rap* fruis combined with *35S::MYB10*, they also turned red (Fig. 5E). The anthocyanin contents in these infiltrated fruit consistently correlated with the color phenotype (Fig. 5F). The expression levels of *MYB10* and *RAP* in these infiltrated fruit were confirmed by qRT-PCR (Fig. 5G). Of note, *RAP* was significantly induced by overexpression of *MYB10* (Fig. 5G, B versus A). Overall, the complementation test proved that *gene31672/GST* is indeed the *RAP* gene.

### Comparison of gene functions between RAP and its paralogs during fruit coloration

**Fig. 6.**
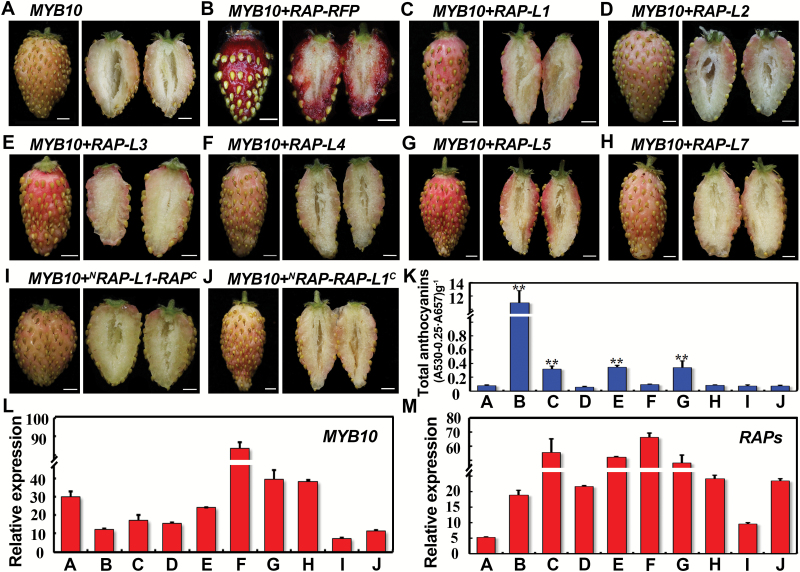
Transient expression of *RAP* and its paralogs in fruit of *rap*. (A) A fruit of *rap* overexpressing *MYB10* by agro-infiltration. (B–J) Fruits of *rap* transiently overexpressing *MYB10* together with *RAP*, *RAP-Like* genes, or chimeric genes between *RAP* and *RAP-L1* (Supplementary Fig. S4B), as indicated. At least 10 fruit were infiltrated for each construct with similar results. (K) Total anthocyanin contents in the fruit shown in (A–J). The values of B–J are compared to A: **, *P*<0.01, Student’s *t*-test. (L) Expression levels of *MYB10* in the fruit shown in (A–J) as analysed by qRT-PCR. (M) Expression levels of *RAP*s in the fruit shown in (A–J) as analysed by qRT-PCR. For (A) and (B), *RAP* was examined; for (C–J), each overexpressed *RAP-Like* gene was examined. *Gene11892* was used as the internal control. Data are means (±SD) obtained from three technical replicates. The experiment was repeated for three times with similar results. Scale bars in (A–J) are 3 mm.

Expression analysis showed that *RAP* was much more abundantly expressed than its other paralogs during fruit ripening in *F. vesca* (Fig. 2D). As RAP and its paralogs share a high level of sequence similarity spanning the entire gene (Supplementary Fig. S4A), it is possible that these *RAP* paralogs have the same anthocyanin binding and transport capacities as RAP. To test this hypothesis, *RAP-L1-5* and *RAP-L7* driven by the 35S constitutive promoter were each infiltrated together with *35S::MYB10* into the *rap* fruit. The protein sequence of RAP-L6 is very similar to that of RAP-L5 (only 9 out of 214 amino acids are different), and hence no results are shown for RAP-L6. *35S::MYB10* only served as the negative control and *35S::RAP-RFP* together with *35S::MYB10* served as the positive control (Fig. 6A, B). RAP-L1, 3, and 5 induced a deeper pink color compared to the negative control, while the other three RAP-Ls had no obvious effect (Fig. 6C–H). Nevertheless, none of the *RAP-Like* genes gave the red color to the same extent as *RAP* (Fig. 6K). To distinguish which part of RAP is more important in determining anthocyanin-binding capacities, the N- and C-terminals were switched between RAP and RAP-L1 (the closest paralog) to create two chimeric proteins (Supplementary Fig. S4B). Transient assays showed that neither of them could promote anthocyanin accumulation (Fig. 6I–K), suggesting that both terminals are necessary for the anthocyanin-binding capacity of RAP. The expression levels of *MYB10* and the *RAP*s were confirmed by qRT-PCR (Fig. 6L, M).

It was shown previously that TT19 is soluble and is localized in the cytosol and on the tonoplast (Sun *et al.*, 2012). To examine the subcellular localization of RAP and its paralogs, *RAP* and *RAP-L1-5* were fused with *GFP* and transiently expressed in tobacco leaves. We found that RAP, RAP-L1, and RAP-L3-5 (data not shown) were localized in the cytosol and nuclei, but they did not overlap with the endoplasmic reticulum marker (Supplementary Fig. S7A, C) (Nelson *et al.*, 2007). In addition, RAP was partially co-localized with the tonoplast marker CBL6-OFP (Supplementary Fig. S7B) (Batistic *et al.*, 2010). Among the proteins examined, RAP-L2 was notable in that it was primarily localized in chloroplasts (Supplementary Fig. S7D). Both the transient functional assay and the subcellular localization highlight the functional divergence of RAP and its paralogs in *F. vesca*.

### Transient knock-down of *RAP* reduces fruit coloration in cultivated strawberry

To test whether *RAP* is essential for fruit coloration in *F. vesca*, transient RNAi was performed to knock-down *RAP* in the Ruegen fruit, and it resulted in white fruit receptacles (Supplementary Fig. S8A). As a control, RNAi of *gene30464*, which encodes a transcription factor not expressed in fruit, did not affect the fruit color (Supplementary Fig. S8B). To determine whether RAP is also important for anthocyanin accumulation in cultivated strawberry (*F. ananassa*), the two constructs were each transiently infiltrated into the fruit of Sweet Charlie, a popular strawberry cultivar. Similar to the results in Ruegen (*F. vesca*), transient knock-down of *RAP* also dramatically reduced fruit coloration in the cultivated strawberry (Supplementary Fig. S8C, D). The anthocyanin content was much lower in *RAP-RNAi* fruit than controls in both the wild and cultivated strawberry (Supplementary Fig. S8E, F), which correlated with significantly reduced *RAP* transcript levels (Supplementary Fig. S8G, H). Therefore, *RAP* is both a good candidate gene for genetic manipulation and a candidate marker for breeding aimed at improving fruit color in cultivated strawberry.

## Discussion

Fruit color is an important trait of fruit quality, and color of foliage is also valuable for ornamental plants. Accumulation of anthocyanins not only gives rise to bright colors in plants, but also benefits human health; hence the regulation of anthocyanin biosynthesis is of great interest. It has been shown that GSTs play important roles in anthocyanin accumulation as a result of the characterization of their loss-of-function mutants in maize, petunia, and Arabidopsis (Marrs *et al.*, 1995; Mueller *et al.*, 2000; Kitamura *et al.*, 2004). Hence considerable efforts have been made in exploring the functions of GSTs with regards to fruit pigmentation in grape, apple, and lychee (Conn *et al.*, 2008; El-Sharkawy *et al.*, 2015; Hu *et al.*, 2016). One GST named *Riant* in peach was found to be responsible for the variegated coloration of petals (Cheng *et al.*, 2015). However, none of these GSTs in fruit crops have been thoroughly studied through genetic approaches, with research being hindered by the difficulties in genetic manipulation of fruit trees. Here, we isolated one green-foliage mutant in wild diploid strawberry, *F. vesca*, which was shown to be caused by a defective *GST* gene. This *rap* mutant is valuable for studying anthocyanin transport in fruit crops.

GSTs form a large gene family. For example, there are 53 GSTs in Arabidopsis (Sappl *et al.*, 2009), 79 in rice (Jain *et al.*, 2010), and 90 in tomato (Islam *et al.*, 2017). GSTs are grouped into several subfamilies. A total of 13 belong to the phi subfamily in Arabidopsis, which is the second most numerous subfamily. Seven *FvGST*s together with *RAP* constitute the phi subfamily in *F. vesca*. Since a single *rap* mutation dramatically lowered the anthocyanin level in the fruit and petiole of *F. vesca*, there is clearly a lack of functional redundancy among the *GST* genes with roles in anthocyanin transportation in strawberry. Our transient expression results using 35S-driven *RAP* and *RAP* family members indicated that the coding sequences are important to their different functions. The differences in coding sequence may also cause distinct subcellular localization that contributes to their functional divergence.

*FvMYB10* codes for a master regulatory transcription factor in anthocyanin biosynthesis in strawberry fruit (Lin-Wang *et al.*, 2010, 2014; Medina-Puche *et al.*, 2014). Recently, one naturally occurring SNP in *FvMYB10* was found to be responsible for the fruit color of different *F. vesca* varieties (Hawkins *et al.*, 2016). Noticeably, both red- and white-fruited *F. vesca* varieties have red petioles, suggesting a minor role of *FvMYB10* in anthocyanin accumulation in petioles. Consistent with this observation, the petioles of H4, YW5AF7, and Ruegen have comparable anthocyanin contents and similar profiles of anthocyanin compounds when examined by HPLC (Xu *et al.*, 2014). Moreover, RNAi mediated knock-down of *FvMYB10* in red-fruited varieties leads to white fruit but does not change the petiole color (Lin-Wang *et al.*, 2014). Taken together, this suggests that *FvMYB10* only plays crucial roles in fruit pigmentation rather than in foliage coloration. The MBW complex is a regulatory paradigm of anthocyanin biosynthesis, and other MYB transcription factors responsible for anthocyanin accumulation in strawberry petioles are yet to be identified.

*RAP* was significantly induced by overexpression of *MYB10* and dramatically reduced in the fruit of *myb10* varieties (YW5AF7 and H4) (Fig. 5, Supplementary Fig. S6; Lin-Wang *et al.*, 2014; Härtl *et al.*, 2017), suggesting that *RAP* expression is regulated by *MYB10*. Interestingly, the *myb10*^−^*rap*^−^ double-mutant infiltrated with *35S::MYB10* still could not develop red color, indicating that the function of *MYB10* in fruit pigmentation depends on wild-type *RAP*. Based on these results, we conclude that not only is *RAP* transcriptionally regulated by *MYB10* but also that it acts downstream of *MYB10* to mediate its effect in fruit coloration. This is consistent with the role of *RAP*/*GST* in pigment transport and that of *MYB10* in pigment gene biosynthesis. Earlier work in lychee showed that *LcGST4* could be up-regulated by *LcMYB1* in the dual luciferase assay in tobacco (Hu *et al.*, 2016). Stronger evidence for a direct transcriptional activation of the GST transporter by *MYB10* requires further experiments, such as testing the binding of MYB10 to the promoter sequence of *RAP* containing the *C1* motif/MYB binding site (TAACTG, Supplementary Fig. S9) (Bodeau and Walbot, 1996).

In this study we found that *RAP* was developmentally regulated at the transcriptional level during fruit coloration. As anthocyanin levels are up-regulated in response to different stresses, the *GST*s involved in anthocyanin accumulation may also be responsive to both internal and external factors. Consistent with this idea, jasmonic acid elicits an obvious increase of anthocyanin content together with induction of GSTs in grape cell suspension cultures (Conn *et al.*, 2008). LcGST4 is significantly induced by ABA treatment and removing fruit from bagging (Hu *et al.*, 2016). In apple, a transcription factor in light signaling (MdHY5) can directly bind to the G-box motif (CACGTG) in the promoter of *MdMYB10* (An *et al.*, 2017), indicating the importance of the G-box in light-mediated regulation of gene expression during anthocyanin production. We found that the promoter of *RAP* also possesses a G-box motif (238 bp upstream of the translational start codon, Supplementary Fig. S9). These findings suggest that *GST*s may be a common downstream target of different regulatory pathways in anthocyanin accumulation.

## Supplementary data

Supplementary data are available at *JXB* online.

Fig. S1. Anthocyanin biosynthetic genes in *F. vesca* and their expression levels in *rap*.

Fig. S2. Integrative Genomics Viewer (IGV; http://software.broadinstitute.org/software/igv/) depiction of the causative SNP of *rap* in *gene31672*.

Fig. S3. Genomic sequences of *RAP* and its paralogs.

Fig. S4. Amino acid sequence alignment of RAP and its paralogs.

Fig. S5. Genotyping of *35S::RAP-RFP* transgenic lines in Arabidopsis *tt19-7*.

Fig. S6. Total anthocyanin contents and expression levels of *RAP* in petioles and fruit of the three *F. vesca* varieties.

Fig. S7. Subcellular localization of RAP and its paralogs in tobacco epidermal cells.

Fig. S8. Transient knock-down of *RAP* reduces fruit coloration in *F. vesca* and *F. ananassa*.

Fig. S9. Promoter sequence of *RAP*.

Table S1. List of primers used for making constructs.

Table S2. List of primers used for qRT-PCR.

Supplementary Figures and Tables

## Author contributions

HL, CK, and ZL conceived and designed the experiments; HL, CD, YL, and JF performed the experiments; CK, ZL, and HL wrote the paper. All the authors have read and approved the paper.
