# Supplementary material for: Reduced Anthocyanins in Petioles codes for a GST anthocyanin transporter that is essential for the foliage and fruit coloration in strawberry
Source: J Exp Bot. 2018 Mar 10;69(10):2595–608. doi: 10.1093/jxb/ery096 (PMC5920330; doi:10.1093/jxb/ery096)
Supplement: Supplementary Figures and Tables [file ery096_suppl_supplementary_figures_and_tables.pdf]

RAP codes for a GST anthocyanin transporter that is essential for the foliage and fruit coloration in strawberry

Huifeng Luo[1], Cheng Dai[2], Yongping Li[1], Jia Feng[1], Zhongchi Liu[1, 3], and Chunying Kang[1*]

1. Key Laboratory of Horticultural Plant Biology (Ministry of Education), College of Horticulture and Forestry Sciences, Huazhong Agricultural University, Wuhan, 430070, China
2. College of Plant Science and Technology, Huazhong Agricultural University, Wuhan, 430070, China
3. Dept. of Cell Biology and Molecular Genetics, University of Maryland, College Park, MD, 20742, USA

*To whom correspondence should be addressed. Tel. +86 13871595425. E-mail: ckang@mail.hzau.edu.cn

SUPPLEMENTARY DATA

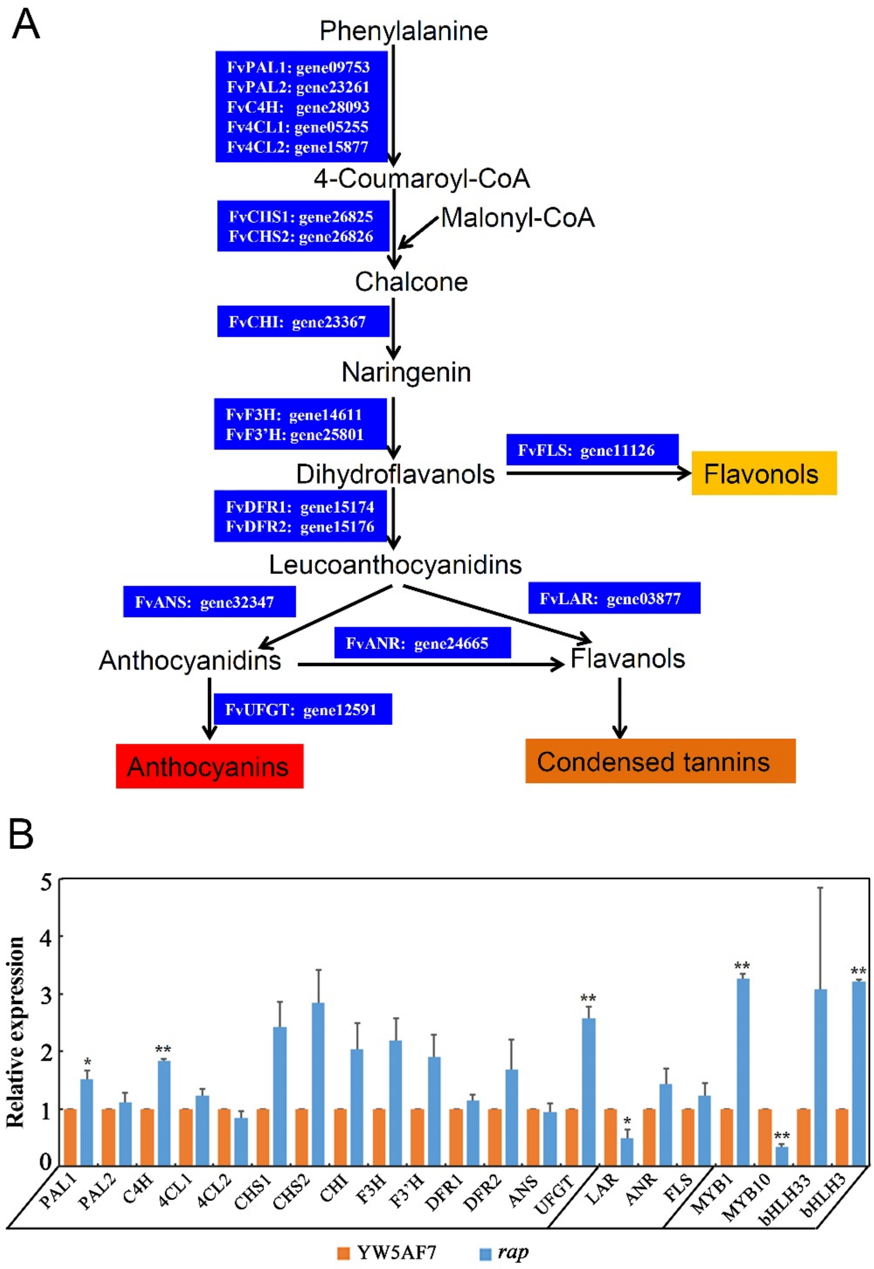

Figure S1. Anthocyanin biosynthetic genes in *F. vesca* and their expression level in *rap*. (A) A diagram showing genes coding for catalytic enzymes in anthocyanin biosynthesis pathway in *F. vesca*. The diagram was adapted from Pillet *et al.*, J Exp Bot, 2015. (B) Expression level of the catalytic enzyme and regulatory genes in anthocyanin biosynthesis pathway in the petioles of YW5AF7 (wild type) and *rap* analyzed by qRT–PCR. Y-axis indicates the relative expression level, which was respectively set to 1 in YW5AF7 for each gene. *Gene11892* was used as the internal control. Data are means±SD obtained from three technical repeats. **, $P < 0.01$, student's *t*-test. The experiment was repeated twice with similar results.

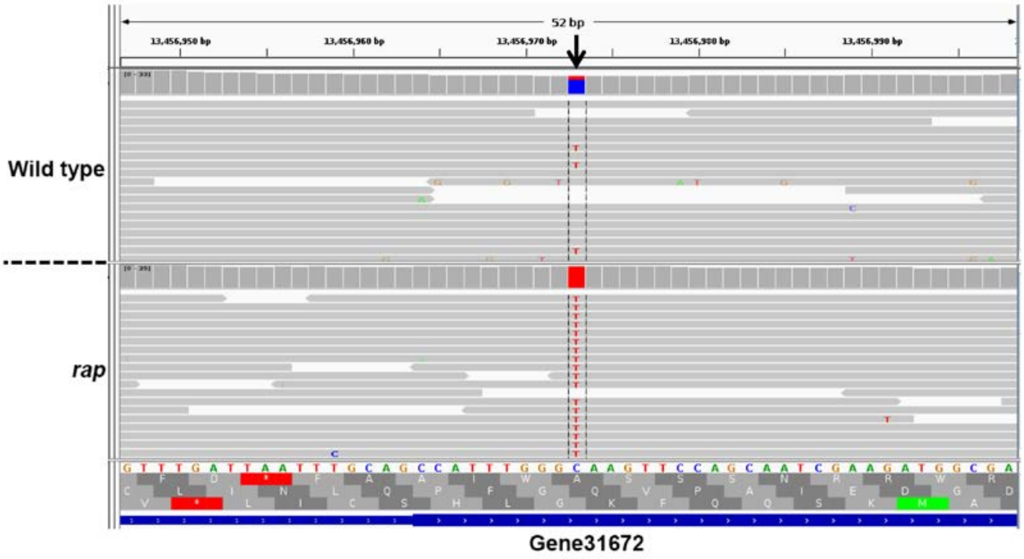

Figure S2. IGV view of the causative SNP of *rap* in gene31672.

Gray bars indicate aligned reads derived from genome resequencing of the wild type and *rap* mutant groups, respectively. Black arrow points to the SNP in the second exon of gene31672, where the wild type C in blue was mutated into T in red.

Figure S3. Genomic sequences of *RAP* and its paralogs.

The genomic sequences from translation start site (ATG) to stop codon for these genes are shown as following. Blue shades indicate exons. Yellow shades indicate introns.

>*RAP*-gene31672

ATGGTACTGAAAGTTTATGGTCCAGTTAGGGCAGCCTGCCCCCAGAGGGTGATGGTTTGCCTTTTGGAGCTAGGGGTTGAGTTT
GAGATTGTGCCTGTTGATCTTCAAGCAGGAGAGCAAAAGCAACCTCACATTCTTGCTCGACAGGTAAATATTAACAGTCTAGC
AACATAATTGCATCAAATTACACACACATACATGTCGGCATATATTTGACTGTTTGATTAATTTGCAGCCATTTGGGCAAGTTCCA
GCAATCGAAGATGGCGATTTCAAGCTTTTTGGTATGGGGGGATGATATGCATACATACATATACTTGTTACTTTCTTAATTTGGGT
CTTTTTTGGAAGTCCATGAAAAAAGTAGTCCTTGCAAAAATGGCAATAACAATATCAGCAGTTGAAATTGTGAACCAAGTCCA
AAATGTTATTGATAATTGGTTATCAAGAGATTAACTAGCTAGGTATAACTAGTATAGTAATACTGTACGTATCTTGGAATAAGATGT
TCAAAGTTCTTGTTGCTGTATGCCTTCATTAACCCGATTTCTGGCTCTCGCTCTGCTACACAGAATCTAGAGCTATCGTAAGATA
CTATGCGGCTAAGTATGCAGAGCGTGGTCCTAACCTGCTAGGAACAACACTGGAGGAGAAGGCACTGGTGGATCAATGGCTCG
AAGTCGAATCACACAACTTCAACGACTTGGTTTTCACTGTGGTACTTCAACTTGTGATCCTTCCCAGCATGGGCCAACCTGGCG
ACTTGGCCTTGGTGCGCTCTTGTGAAGAAAAACTGAAAAAGGTATTCGATGTGTATGAGGAGAGACTGTCCAAGAGCACCTAT
CTGGCCGGAAACTACTTCAGTTTGGCTGATCTGAGCCATCTTCCGGCGATTCGGTTTCTGGTTGACGAGTTCAAAATGGGACAT
TTGATCACGGAGAGGAAGAATGTGAATGCTTGGTGGAAAGATATTTCCAATAGGCCTGCATGGAAGAAACTTATGAAGCTTGC
TCAATACTAG

>*RAP-L1*-gene28763

ATGGTGGTAAAGGTGTATGGCCCCTCATATGCTTCTCCTAAACGAGTTCTAGTGTGTCTTGTTGAGAAGGAGATTGAGTTTGAA
ACCATCCCCATTGATCTTTTCAAGGGAGAACATAAAGATCCTGAATTCCTCAAATTACAAGTAATCACTTTGTGATTCATATCCAT
CATCTATTCTATATCTGTTTGTTTACTTGGTGGTTGATCGTTTGCATTGTCTTCTCTTTTCATGTAGCCCTTTGGAGCAGTTCCTGT
TATTCAAGATGGGGATTATACTTTATATGGTATCTACTTGAAATGAGCTTTCATGTACTGTTTTGATTTATTGTTGTTGTTCATGTG
CTTAATATACTTATTTGAATATGTAGTTCAGATTGAGTGACCATTTATTTCCCTTTCTGGTTGCCTCCTGAATTTGCAGAAGTTTAG
AAAAACAGTTATACCAAGATTTTTAAGATACAACTCAATTATTTTCATCGTAACAAGACATAGCATTCCATATCAGAGGGGAACT
ATAAGTTCTTTTGGTTTAACAAGACGTAGCATTTCATATCAGTGGTTTTACTTAACTTTTTATTTGAATTATCTGGAATATCAAATT
AGCATTGGAATGTTTTTCTGATAGACTACTTTAGTTCCTTTCAATCCTAATTTATTGAATAAAGTCATTCAAATTTGATTTTGCAG
AATCACGGGCAATTATAAGATACTACGCAGAAAAGTACAAGTCACAAGGGACTGATTTGCTAGGAAAGACGATTGAGGAAAG
AGGCCTTGTGGAACAATGGCTAGAGGTTGAAGCTCAAAACTACCACCCATCAATTGACAACTTGGTCATTCACATTTTGTTTGC
CCCAGTCCAGGGTTTCCCTTCAGACCCAAAGATAATTCAAGAGAGTGAAGAGAAGTTAGGCAAGGTGTTGGATGTTTATGAGG
AGAGGCTATCAAAGAGCAAATATTTGGCTGGTGATTTTTTCAGCCTTGCTGATCTTAGCCACCTTCCATTTACACAGTACTTGGT
GGGTGCTGTAGGGAAAGACTACATGATAAAGAGCAGAAAGCATGTCTCAGCTTGGTGGGATGATATTAGCAACAGGCCATCGT
GGAAGAAGGTGCTCGAGTTCGGTGCCCCATTTTAA

>*RAP-L2*-gene08595

ATGGCGACTCCGGTGAAAGTGTACGGGCCACCGATATCCACCTGCGTGTCGAGGGTGTTGGCCTGTCTCCTTGAGAAAGAAGT
GCCCTATCAGCTCATTCCGGTCAACATGGCCAAAGGCGAGCACAAGAAGCCTGCATACCTCAAGATCCAGGTATCTTTATACTC
CCACATTTACAATGAACATGATTCTGTTGAATTCTCATTGTTTTTCTTTTCAACTGTAACAGCCATTTGGACAAGTCCCGGGTTTT
GAAGACGATGGCTTCACCGTGTTCGGTATGTATATATATGCTTCAATGCATGGAATTAGATTGTGGATGTGGGGTTGATTTGTTCT
CTACATGTCTTGGTAGTAATGTGATTTGTGATTTCAGAATCAAGGTCGATATGCCGGTACATATGTGATAAGTATGCAAAGCAGG
GAAACAAGGGCTTGTATGGGGACAACCCATTGGCAAAGGCCTCCATAGATCAGTGGCTAGAAGCCGAAGGACAAAGCTTCAG
CCCTCCAAGCTCGGTTCTGGTGTTCCAGCTTGCATTCGCGCCAAGAATGAAGCTGAAGCAGGATCAAGTGGCGATCAGGCAGA
ACGAAGAGAAGCTGAAGAAGGTGCTGGACGTGTACGAAGAGAGGCTAAGTGAGAGCCGGTTCTTGGCGGGTGACGAGTTCA
CTCTAGCCGACCTTTCTCACTTGCCCAACACACACTACTTGGTCAATGCAACAGATATAGGAGAGCTGTTTACAGAGAGAGAAT
GTGTGGGCAGGTGGTGGGCTGAGATATCTAGCCGAGAGTCGTGGAAGAAGGTGGTTGAAATGCAGAAGCCTTGA

>*RAP-L3*-gene22014

ATGGGACTCAAGCTTCATGGCCTTGCTCTCTCAACATGCACTGCTCGTGTCACCACCGCCATTTACGAGAAAGGGCAGGAATT
CGAGCTCATCCCTGTTAACCTTACCACCGGAGAACACAAGCAGTCAGGTTTCCTTGCCAAAAATGTAAGAACATCATGTAACAT
TATGTCTGCATATATATTTTCCGACAAGGTAAGCTCTTGATCCAATCTGACATGGTGTTGTGTTTGTGTTGATGATATTGCAGCCC
TTTGGTCAAATTCCAGCCCTAGAGGATGGTGATCTCACTCTTTTTGGTAATCTTTCCTTTCCCTATTGCAGTGATTTTAAGAATTT
CATATATTAGAAGATGATGACAATTCAGAACAAGCTAGCATGGATTGGCCAAAAGTAGCAACATTAATGAATACTCTCATCCGGC
CATGAAAATGTGATTAATTTCCGGCAAACATACCAGAATAAAATTCAAAACCTGTTTTAGGAGTAATCTGGTCTTTTTGGCCTAG
TGATACAATTCATATGATTTTAGATTGGTTTGAAAATATTGAAGTATTTGAGGTATGATAACTGTAGTATATGTTTGTAGCTTTTAC
TATCCATGCATACTCAGTTAGCATATCTGTGGAATGAACATTGACAGAGTCTAGAGCCATTGCATCATATGTTGCCGAAAAGTAC
AAGGACTCTGGTTCTGATCTCATCAGGCACAACAACCTCAAGGAAGCTGCACTTGTCAAGGTGTGGTCGGAGGTGGAGGCTC
AGACCTTCCACCCGGTAATCTCCACCATTGTCCACCAGCATCTCATTCTCCCATTGTTTGGACAGCAGCCAGACCAAGCAGTGA
TCGATGCAAACCTGGAGAAGCTCAAGAACATCCTCAATGTCTATGACGCGAGGTTGAAGAGCAGCAAGTACTTGGCAGGAGA
TTTCTTCAGCATCGCGGATCTTAACCACTTTCCCTACCTTTTCTACTTCATGAGGAGCCCTTGGACTTCGGTGGTGAATGATCGT
CCTCATGTGAAAGCCTGGTGGGAGGACATTTCTTCAAGGCCAAGCTTCAAGAAAGTAGCTGAGGGAATGGCCTTAGGTCAGA
AGTAG

>*RAP-L4*-gene10549

ATGGTGCTCAAGCTGCATGGACTTTCTGTGTCGACCAACACAGCTCGTGTCGTGGCTTGTCTACATGAGAAAGATGTCGATTTC
CAGCTTGTCCCGGTTGATCTCTTTGCCCGTGGAAACAAGGAGCCTTCTTTTCTTGCCAAGAATGTAAGTTTCTTTCCCATGCTT

GTATTACAAGCTTATAATTTCATTCTATCATCATATAAGCTCATTTCGACTGTTAGCAGTCGTTTAATTCGTATATATGTTTGTTCTC
AATCCTCTATCTTGCAGCCCTTTGGCCAGATTCCAGTATTAGAAGATGGTGACATCACTCTTTTTGGTAAGCATGTTAGATTACTT
CTTCATTATTTGATAAATTTCAAAATTTGATGACTTAGATATACAACTAATTTTGTCTCACCATCTGTTTGATTTTTGTTACACCAG
AATCTAGGGCAATCTCATCCTACATAGCAGAAAAATTCAAGGAAACCGGAACCGATCTCATACGGCACGAAAGCTTCAGTGAA
GCTGCACTGGTGAAGGTTTGGACAGAAGTTGAATCCCAACAATACCACCCAGCAATCTGTCCCATTATATACGAGTTTTTCGGA
AAGCCAGTGCAAGGCTTGGAACCAGACCAGGCAGTGATAGATGCAAATTTGGAGAAGCTAGAGATGGTGCTCGATGTTTATGA
GGCCAAGCTGAGCAGCACCAAGTACCTTGCTGGTGATTTCTACAGCCTAGCCGATCTTCACCATTTCCCTTACACTTTTTACTTC
ATGAAGACTCCTTTGGGCTCAGTGATCAGTGATCGTCCTCATGTTAAGGCCTGGTGGGATGACATTACAGCTAGGCCTGCTTTG
AAGAAAGTTGCTGAGGGTATGACTTTCGGTGAGAATCAGTGA

>*RAP-L5*-gene10550

ATGGCCGGCATCAAAGTCCATGGAACCGCTATGTCAACTGCAGCAGCGCGAGTTTTGGCTACCCTCTACGAGAAAGAGGTGGA
ATTTGAGCTTGTTCCGATAGACATGAGAGCCGGTGAACATAAAAAGGACTCCTTCCTTGCCCTCAATGTAAGTTTCTTGAAATG
AAAACAGAGTTGCTTTTGGCATGTTTTCTCTTTGATTCTTACCTCTTAATTTGTTTGTAATTCAATCCAGCCATTTGGTCAAGTTC
CAGCTTTTGAAGACGGAGATCTTCAGCTCTTTGGTAAGTTTCTGCTTAGCTCAGCCACATCAAATTCACTCAGCATATATAAAAT
TGTGTTTGCGACTTTGTATGATTGTATCTAAGATTCTAAGATGCGAACCTTGTTGAATGCAGAGTCAAGAGCAATTACACAATAC
ATTGCCCATGAGTATGCTTCAAAGGGAACCCCGCTGATAGTCCCAGACTCGAAGAAGATGGCAATTCTATCAGTGTGGACAGA
GGTGGAGGCACAAAAGTTCGACCCTGCAGCCTCAAAACTGACATATGAGCTAGTAATAAAACCTCTCTATGGCACGGTCACTG
ACTTCGCAGTCGTGGAGGAATTCGAAGCTAAGTTGGGTACAGTTCTCGATGTCTATGAGAAACGTCTGGGACAATCGAAATAC
TTGGGAGGTGATTGCTTCAGCTTGGCAGATCTTCACCACCTTCCAACTACACAATACTTGATGGGAACACAAGCCAAGAAGCT
CTTTGAGTGCCGCCCGAATGTTAGCGCATGGGTAGCTGATATCACTGCCAGGCCTGCTTGGACTAAAGTCCTCGCCATGAGAGC
TCACTAA

>*RAP-L6*-gene10551

ATGGCCGGCATCAAAGTCCATGGAACCGTTTTCTCTACTGCAGCAGCGCGAGTTTTGGCTACCCTCTACGAGAAAGAGGTGGA
ATTTGAGCTTGTTCCGATAGACATGAAAGCCGGTGAACATAAGAAGGAGTCCTTCCTTGCCCTCAATGTAAGTTTCTTGAAATG
AAAACAGTTGCTTTTGGCATGTTTTCTCCTTGATGTTCTTACCCTTCTTAATTTGTTTGTAATTCAACCCAGCCATTTGGTCAAGT
TCCAGCTTTTGAAGACGGAGATCTTCAGCTCTTTGGTAAGTTTCTGTTTAGCTCAGCCACATCAAATTCACACAGCACATGTATA
TTGTTTGTGACTTTGTATCTAAGATGCGAACCTTGTTGAATGCAGAGTCAAGAGCAATTACACAATACATTGCCCATGAGTACG
CTTCAAAGGGAACCCCGCTGATAGTCCCAGACTCGAAGAAGATGGCAATTCTATCAGTGTGGACAGAGGTGGAGGCACACAA
GTTCGACCCTGCAGCCTCAAAACTGTCATATGAGCTAGCAATAAAGCCTCTCCTTGGCATGGTCACTGACTTCGCAGTCGTGGA
GGAATTCGAAGCTAAGTTGGGTACAGTTCTCGATGTCTATGAGAAACGTCTGGGACAATCGAAATATTTGGGAGGTGATTGCTT
CAGCTTGGCAGATCTTCACCACCTTCCAACTACACAATACTTGATGGGAACACAAGCCAAGAAGCTCTTTGAGTGCCGTCCGA
ATGTTAGCGCATGGGTAGCTGATATCACTGCCAGGCCTGCTTGGACTAAAGTCCTCGCCATGAGAGCTCACTAA

>*RAP-L7*-gene10552

ATGGCAGCAGAAGGCAGAAAGCTGTACGGCGACCTGAACACACCGGAAACCTTGAAGGTTGTGGCATGCCTGTTCGAACATG
ATCTTGATTTTGAGTTTGTTCCAATCAATCTTGAAGCCAACCAACACAGGGAAAAACAATTTCTTTCTATGAGTGTAAGTACTTA
ATTGACTATATAATACTTATAATCATGCATTTTCTTAAATATGTATCCATTATTCTGATATAATCAATGAAATATAACTGGATCGTGTA
GCCCTTTGGTAAATTGCCTGTCTTGGAAGATTTGGTAAGCATATGCATTCAATCACTTTGGACTCTGATGAAATTAAGTTGTTGA
TTAATTCTGATAAATTGGTAGTTAAAGCCTTAGTTTACGTTCCCGGCCAGTAATGAAGTAATATGTTGTTGCAGAAGCCACAACG
ATAGTAAGATACATGGGTCATATGTATGCAAATAAGAAGAAAGTGGGAGAAGAGCTTATCTTCTGGGAGTCACATAAACAAGCC
TTGGTGGCTAATTGGATTGATGTGGAAGACCACTACTTTGAACCACCAGCACTCAAGCTCATATCGTTGTTGTCAAGTAACAAG
GCTATCGACGATGTCGGCGAGTCCAATGGTGTTGATCGTGTAATAGATGAACTGAGCCAAGTTCTGGATGTGTACGAGGCCCAG
CTGGGGAGGTTCAAGTACCTGGCGAGTGACAAGTTCACCGTTGCTGATGTTCTTCACCTCCCAAACTTGCACAGCCTTATGGG
AATCGCCTCGACTAAGAAGCTCATAGATTCGCGTCCTCATGTCAGTGCTTGGTGCTCTGAGATTCTTGCTCGCCCTTCATGGACC
AAGGTGCTTCAGATGAAGAAACAGAAGATGATCTCTCTAGCCTAG

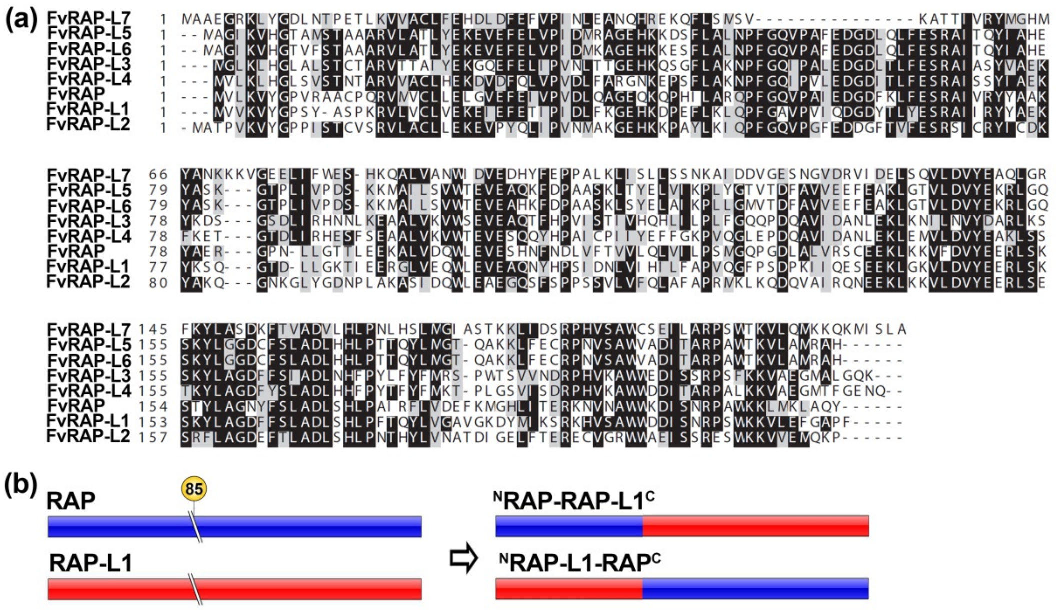

Figure S4. Amino acid sequence alignment of RAP and its paralogs.

(A) Amino acid sequence alignment of RAP and its 7 paralogs in *F. vesca*. The number before each row represents the position of the first amino acid. Identical amino acids are highlighted with black, while conservative substitutions are marked with gray. (B) Design of the chimeric proteins between RAP and RAP-L1 shown in Fig. 6. The length of the two proteins is exactly the same. Their N- and C-termini are switched. N-terminus is from the 1st to the 85th amino acid, while C-terminus is from the 86th to the last amino acid.

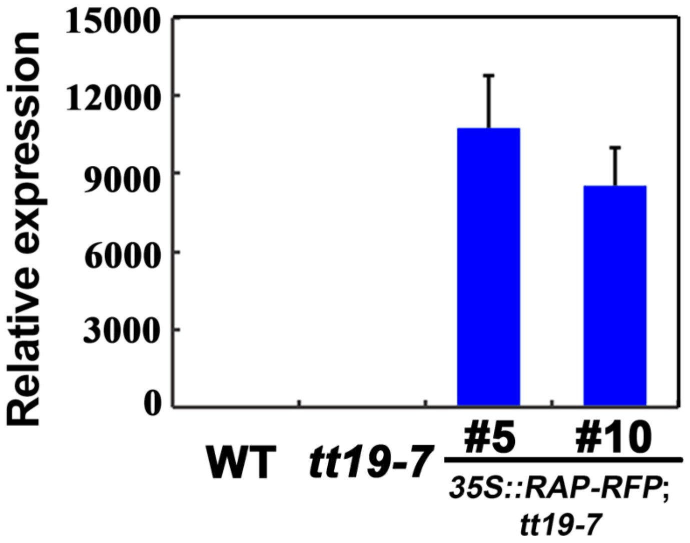

Figure S5. Genotyping of the *35S::RAP-RFP* transgenic lines in *Arabidopsis tt19-7*.
Relative expression level of *RAP* in leaves of WT, *tt19-7*, and two transgenic lines of *35S::RAP-RFP* (L5, L10) in the *tt19-7* background was shown examined by qRT-PCR. *Actin2* (At3g18780) was used as the internal control. Data are means ± SD obtained from three technical repeats.

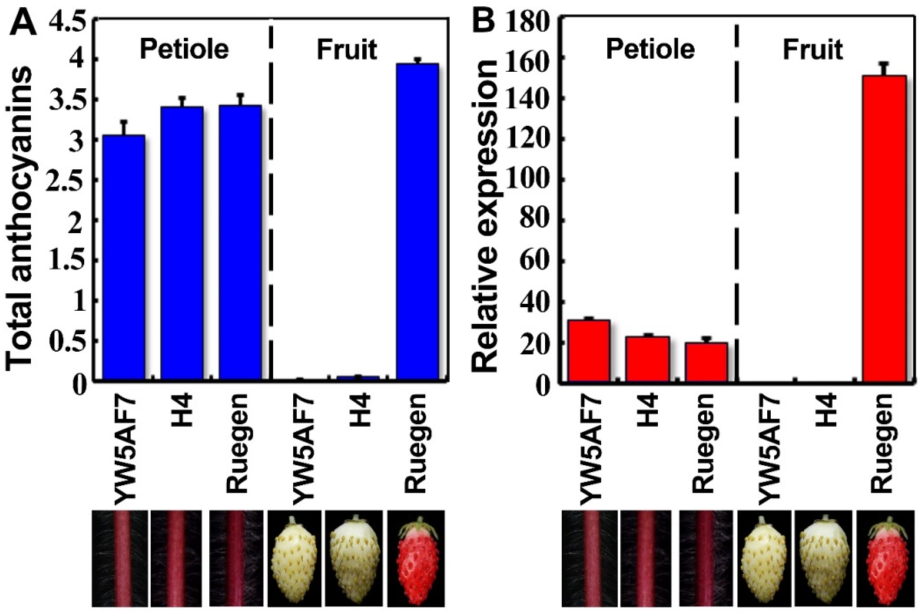

Figure S6. Total anthocyanin contents and expression level of *RAP* in petioles and fruits of the three *F. vesca* varieties.

(A) Total anthocyanin contents in the petioles of mature leaves and the mature fruits of *F. vesca* varieties YW5AF7, H4, and Ruegen, respectively. The unit of total anthocyanin contents in Y-axis is (A530-0.25·A657) g^{-1}. (B) Expression level of *RAP* in the petioles of mature leaves and the mature fruits of *F. vesca* varieties YW5AF7, H4, and Ruegen, respectively. *Gene11892* was used as the internal control. Data are means±SD obtained from three technical repeats. The experiment was repeated twice with similar results.

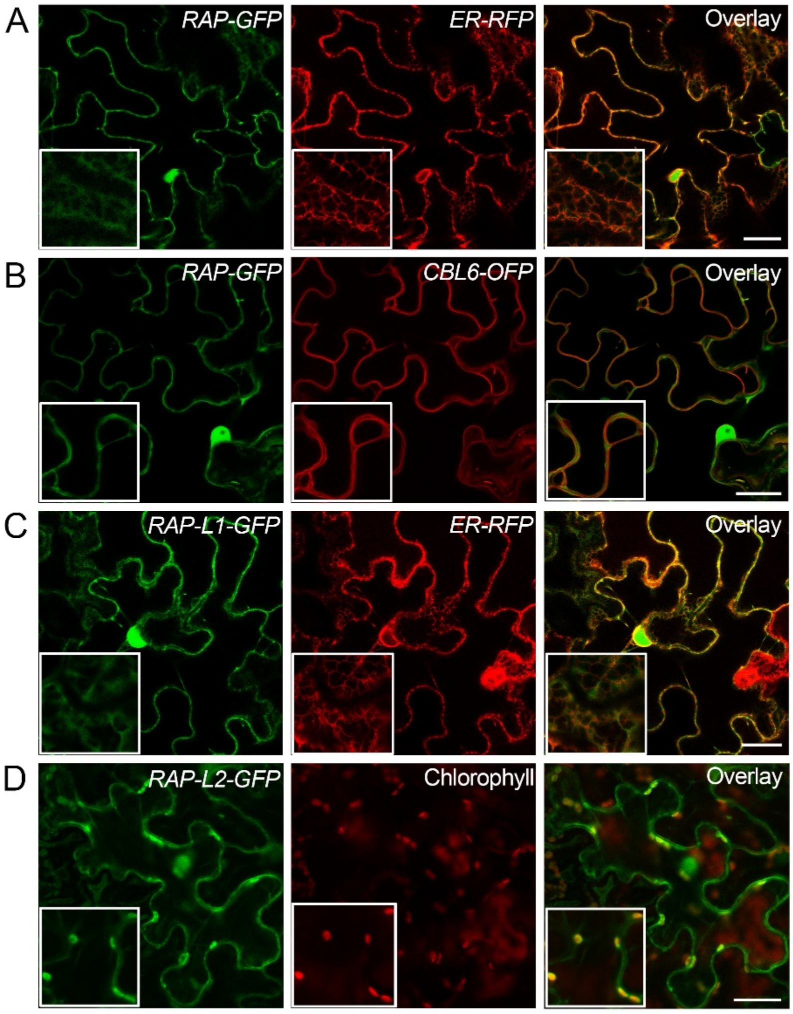

Figure S7. Subcellular localization of RAP and its paralogs in tobacco epidermal cells. (A) and (B) Confocal images of *RAP-GFP* (green) co-expressed with *ER marker-RFP* (red) or tonoplast marker *CBL6-OFP* in tobacco epidermal cells, respectively. (C) Confocal images of *RAP-L1-GFP* (green) co-expressed with *ER marker-RFP* (red) in tobacco epidermal cells. (D) Confocal images of *RAP-L2-GFP* (green) with chlorophyll autofluorescence (red). Inset is the enlarged image of either the same or a different cell. Bar =25 μm.

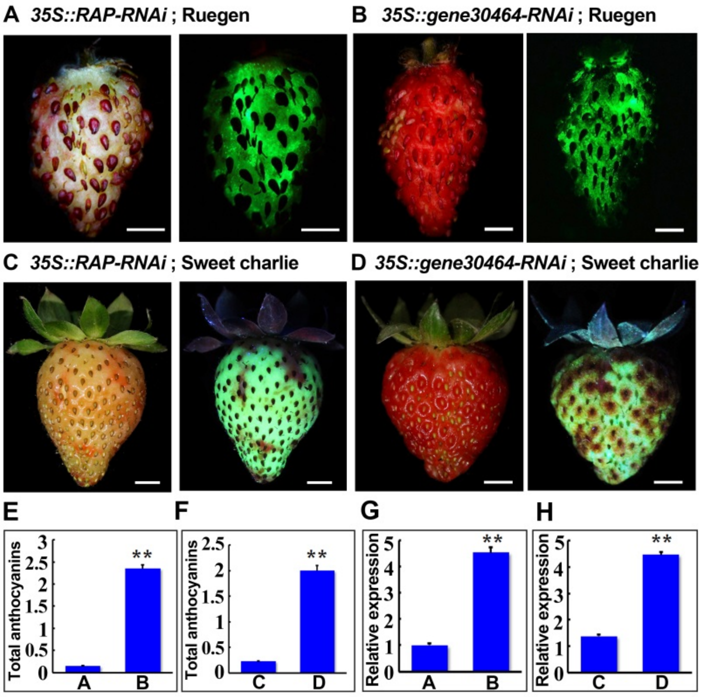

Figure S8. Transient knock-down of *RAP* reduces fruit coloration in *F. vesca* and *F. ananassa*.

(A) One fruit of Ruegen infiltrated with *35S::RAP-RNAi*. (B) One fruit of Ruegen infiltrated with *35S::gene30464-RNAi* (control). (C) One fruit of Sweet Charlie infiltrated with *35S::RAP-RNAi*. (D) One fruit of Sweet Charlie infiltrated with *35S::gene30464-RNAi* (control). At least 10 fruits were used for each construct with similar results. The green fluorescence is from the *35S::GFP* cassette in the vector. (E) and (F) Total anthocyanin contents in the fruits shown in (A) to (D). The unit of total anthocyanin contents in Y-axis is (A530-0.25·A657) g^{-1}. (G) and (H) Expression level of *RAP* in the fruits shown in (A) to (D) examined by qRT-PCR. *Gene11892* was used as the internal control. Data are means ± SD obtained from three technical replicates. **, $P < 0.01$, student's *t*-test. The experiment was repeated for three times with similar results. Bar = 3 mm in (A) and (B). Bar = 5 mm in (C) and (D).

Figure S9. Promoter sequence of *RAP*.

>Promoter of *RAP*-gene31672 (1,114 bp)

Green shade indicates C1 motif/MYB binding site. Yellow shade indicates R motif/G-box.

TTTAGACCGCCCTATCATTATTCAATCCTCTACAGTTCCATGCCAAGATCTCCATGAACAACCTAGAATCGATGCCAAAACGAAA
CTCTAGCAGAGCACAGCGTTTTCTATACTCGATGCCAAGCTTTAACTAATTAACTTAAAAATACACAATGGCTTTGGGAAAAGC
CCATTGGACTATATAGAGCCCAAGCTAGCCTATTTAATGTTATTGTAGTCTTTGAAATGATCTCAGCATGCACTTTGATGCATTTA
CCACTCGCTCATTGTCCAAATTAATGGAGGTTTTCAGGTGGCTATATTATGGTTTTTCAGTGGTACCTTTTCACAACTAT**TAACTG**
GTACGCACATTTGGTTTGCTTCTCATGAATCCAAGCTAGGGGGCAATATATACGTACGAAATGCAATTCTCTTCTCATGAAGATG
AAGATGAAGATGAAGGTAATCGGGTAACACACGTACTCTTTGCACATAATGCAATTTGCTTCTTATAAATGCAACACATACACTG
TGGCTCACACAAAAGTGCCATCTATTTTGACATTGTCAATGATTAATTACGTGTATATTTTTCATCCAAGACATATATATATATAGGTG
AGTAAGGCTTTAGCTTATTGAGTCTTGCATGTGATCTGCCGGGCGGCCGGGCATGTGCTAACCCCCAAGGCTAAGGCCACTATT
CGTAACAAATACCTAATTCGATTAATTAGTAGTGATCGAGTGAATGAATCTCTGTCGACGACGTATTCCTATACCATCCACTCCTG
ATTTTTGTCCCTTTTCTTTCTCTTGTTGTGAGGTGTGACAAGTACATTATACCAGTACGTCGTCCTTATAAATTGGCATATGTATGA
GTATGGCTGGCTCAAACTAGT**CACGTG**TGGAAGGTGGCAACCCCCGGGTTGTTCATCATCTTGGTAATAATAAGGTCCCATATA
TGCTTTCAATTAGTAATATATAGTGGCCATCTGATAGCTGCTTCAGTGCTTCATGCATACCTCTATATATATATAAGCGAGACTAAA
CCCCCAAACAATCCCAATACAGCTACTGAACGATTTCTTGTGCTCTGATACTACTTATTACCGGCCAGTTTTTCTTTACAGCTCG
CAG

Table S1. The list of primers used for making constructs.

Construct	Primer name	Primer sequence (5'-3')
gRAP::RAP	B1-Gene31672-co-F	GGGGACAAGTTTGTACAAAAAAGCAGGCTTTTAGACCGCCCTATCATTATTCA
	B2-Gene31672-co-R	GGGGACCACTTTGTACAAGAAAGCTGGGTGAATTCACATAGTAGAGTACTTAC
35S::RAP-RFP	B1-Gene31672-F	GGGGACAAGTTTGTACAAAAAAGCAGGCTATGGTACTGAAAGTTTATGGT
	Gene31672-RFP-R	AGGAGGCCATGTATTGAGCAAGCTTCATAAGT
	Gene31672-RFP-F	TGCTCAATACATGGCCTCCTCCGAGGACGTC
	B2-RFP-R	GGGGACCACTTTGTACAAGAAAGCTGGGTTTAGGCGCCGGTGGAGTGGCGC
35S::RAP-RNAi	B1-Gene31672-RNi-F	GGGGACAAGTTTGTACAAAAAAGCAGGCTACAGCTCGCAGATGGTACTGAAAG
	B2-Gene31672-RNi-R	GGGGACCACTTTGTACAAGAAAGCTGGGTGGAAGATGGCTCAGATCAGCCAAA
35S::RAP-L1	B1-Gene28763-ox-F	GGGGACAAGTTTGTACAAAAAAGCAGGCTATGGTGGTAAAGGTGTATGGCCCCT
	B2-Gene28763-ox-R	GGGGACCACTTTGTACAAGAAAGCTGGGTTTAAAATGGGGCACCGAACTCGAG
35S::RAP-L2	L1-EcoRI- gene08595-F	ACTGGATCCGGTACCGAATTCATGGCGACTCCGGTGAAAGTGTAC
	L2-EcoRI-gene08595-R	CTCGAGTGCGGCCGCGAATTCTCAAGGCTTCTGCATTTCAACCAC
35S::RAP-L3	B1-Gene22014-ox-F	GGGGACAAGTTTGTACAAAAAAGCAGGCTATGGGACTCAAGCTTCATGGCCTT
	B2-Gene22014-ox-R	GGGGACCACTTTGTACAAGAAAGCTGGGTCTACTTCTGACCTAAGGCCATTCCC
35S::RAP-L4	B1-Gene10549-ox-F	GGGGACAAGTTTGTACAAAAAAGCAGGCTATGGTGCTCAAGCTGCATGGACTT
	B2-Gene10549-ox-R	GGGGACCACTTTGTACAAGAAAGCTGGGTTCACTGATTCTCACCGAAAGTCAT
35S::RAP-L5	B1-Gene10551-ox-F	GGGGACAAGTTTGTACAAAAAAGCAGGCTATGGCCGGCATCAAAGTCCATGGAA
	B2-Gene10551-ox-R	GGGGACCACTTTGTACAAGAAAGCTGGGTTTAGTGAGCTCTCATGGCGAGGACT
35S::RAP-L7	L1-EcoRI-gene10552-F	ACTGGATCCGGTACCGAATTCATGGCAGCAGAAGGCAGAAAGCTG
	L2-EcoRI-gene10552-R	CTCGAGTGCGGCCGCGAATTCCTAGGCTAGAGAGATCATCTTCTG
35S::MYB10	B1-Gene31413-ox-F	GGGGACAAGTTTGTACAAAAAAGCAGGCTATGGAGGGTTATTTCGGTGTGAGA
	B2-Gene31413-ox-R	GGGGACCACTTTGTACAAGAAAGCTGGGTTCATACGTAGGAGATGTTGACTAGATCA
35S::RAP-RAP-L1	B1-Gene31672-F	GGGGACAAGTTTGTACAAAAAAGCAGGCTATGGTACTGAAAGTTTATGGT
	31672-28763-R	TCGTCTTTCCCAGGTTAGGACCACGCTCTG
	31672-28763-F	TCCTAACCTGGGAAAGACGATTGAGGAAAG
	B2-Gene28763-ox-R	GGGGACCACTTTGTACAAGAAAGCTGGGTTTAAAATGGGGCACCGAACTCGAG
35S::RAP-L1-RAP	B1-Gene28763-ox-F	GGGGACAAGTTTGTACAAAAAAGCAGGCTATGGTGGTAAAGGTGTATGGCCCCT
	28763-31672-R	TTGTTCCTAGTAGCAAATCAGTCCCTTGTG
	28763-31672-F	TGATTTGCTACTAGGAACAACACTGGAGGA
	B2-Gene31672-R	GGGGACCACTTTGTACAAGAAAGCTGGGTCTAGTATTGAGCAAGCTTCATAAG
35S::RAP-GFP	P1-gene31672-NdeI-F	TCTTCACTGTTGATACATATGATGGTACTGAAAGTTTATGGT
	P2-gene31672- EcoRI -R	GCCCTTGCTCACCATGAATTCGTATTGAGCAAGCTTCATAAGT
35S::RAP-L5-GFP	P1-gene10550-NdeI-F	TCTTCACTGTTGATACATATGATGGCCGGCATCAAAGTCCATGGAA
	P2-gene10550- EcoRI -R	GCCCTTGCTCACCATGAATTCGTGAGCTCTCATGGCGAGGACTT
35S::RAP-L2-GFP	P1-gene08595-NdeI-F	TCTTCACTGTTGATACATATGATGGCGACTCCGGTGAAAGTGTAC
	P2-gene08595-EcoRI -R	GCCCTTGCTCACCATGAATTC AGGCTTCTGCATTTCAACCACCT
35S::RAP-L1-GFP	P1-Gene28763-NdeI -F	TCTTCACTGTTGATACATATGATGGTGGTAAAGGTGTATGGCCCCT
	P2-Gene28763 EcoRI -R	GCCCTTGCTCACCATGAATTCAAATGGGGCACCGAACTCGAGCAC
35S::RAP-L3-GFP	P1-Gene22014-NdeI -F：	TCTTCACTGTTGATACATATGATGGGACTCAAGCTTCATGGCC
	P2-Gene22014- EcoRI-R：	GCCCTTGCTCACCATGAATTCCTTCTGACCTAAGGCCATTCCCT
35S::RAP-L4-GFP	P1-Gene10549-NdeI -F：	TCTTCACTGTTGATACATATGATGGTGCTCAAGCTGCATGGACTT
	P2-Gene10549- EcoRI-R：	GCCCTTGCTCACCATGAATTCCTGATTCTCACCGAAAGTCATACC

Table S2. The list of primers used for qRT-PCR.

Gene name	Gene ID	Direction	Primer sequence (5'-3')	Amplicon Size
FvPAL1	gene09753	Forward	GGATCCAAAACGGTTCCTTGGACA	248bp
		Reverse	GGCAGACTCATCCAGTTCAACCTT	
FvPAL2	gene23261	Forward	TGGCGCTTAACGGAAACGGAAA	166bp
		Reverse	GGCTTCCGGTAGTCATTCACCATT	
FvC4H	gene28093	Forward	ACTTTATTCCCGTGCTCC	212bp
		Reverse	GACATTGTCCTCGTTGAT	
Fv4CL1	gene05255	Forward	CTCAAACAGCCTACGTCGAA	113bp
		Reverse	CTCGGAGAAATGCAACACAT	
Fv4CL2	gene15877	Forward	GAGGCCACAAGCACAACG	245bp
		Reverse	CTCACCACAAAGGCAACC	
FvCHS1	gene26825	Forward	AAGTCTGCAGCTAATGGGCACAAG	145bp
		Reverse	ACTCGAATGAACCCGGATGCCTTA	
FvCHS2	gene26826	Forward	GAGACCGTTGTGCTTCACAG	149bp
		Reverse	GAATGCCCAAATGAAGATCA	
FvCHI	gene23367	Forward	GTCGTTTCCTCCCTCCG	172bp
		Reverse	CGTCTTGCCCTTCCAC	
FvF3'H	gene25801	Forward	TCACGACGCTAACTTCTC	231bp
		Reverse	TTCACTACCTTTGACCCT	
FvF3H	gene14611	Forward	TGTCCATAGCGACATTCCAGAACC	188bp
		Reverse	CTCTGAATCCTGCAGTTGCTCCTT	
FvDFR1	gene15174	Forward	CACGATTCACGACATTGCGAAATT	139bp
		Reverse	GAACTCAAACCCCATCTCTTTCAGCTT	
FvDFR2	gene15176	Forward	CCGGACTTTCGCCTCTATTT	118bp
		Reverse	GCTTTCGGATGCTCGTACA	
FvANS	gene32347	Forward	GAAGTGCGTACCCAACTCCATCGT	117bp
		Reverse	ACCTTCTCCTTGTTGACGAGCCC	
FvUFGT	gene12591	Forward	TTTGGTTCGGTGCTCATA	265bp
		Reverse	AGCATCTGTCCCATCTGGT	
FvLAR	gene03877	Forward	ACAGAGGCGCCATCATCCTC	233bp
		Reverse	CATGGTCAGACCCGGTTCCA	
FvANR	gene24665	Forward	TGAAGGGAGCTTTGATGCTGCT	120bp
		Reverse	ACTCCTTGGACTCCTGGCTTG	
FvFLS	gene11126	Forward	TGATCTTGCTCTTGGTGTGGTT	150bp
		Reverse	TCACCAATGTGGATGACTAGGG	
FvMYB1	gene09407	Forward	ATGAGGAAGCCCTGCTGCGA	235bp
		Reverse	AACGACGCAACCCTGCAGCC	
FvMYB10	gene31413	Forward	TGGCATCATGTTCCTCTCAAA	235bp
		Reverse	CTCTGCAAACTCTCCTCTCTTG	
FvbHLH3	gene27827	Forward	TGACGACGCCTCCAACAACT	228bp
		Reverse	GGTGGAGACCGTTTCCGAGT	
FvbHLH33	gene19321	Forward	AATCTTCCTCTGCTCCTC	169bp
		Reverse	TCTTCATCATGTCCACGT	
FvRAP	gene31672	Forward	CAAGTTCCAGCAATCGAAGA	211bp
		Reverse	TGGGAAGGATCACAAGTTGA	
FvRAP-L1	gene28763	Forward	ACAACCCTTTGGAGCAGTTC	200bp
		Reverse	CCAAGTTGTCAATTGATGGG	
FvRAP-L2	gene08595	Forward	CAAGAAGCCTGCATACCTCA	131bp
		Reverse	TGTTTCCCTGCTTTGCATAC	
FvRAP-L3	gene22014	Forward	CTCTCTCAACATGCACTGCTC	148bp
		Reverse	TCTAGGGCTGGAATTTGACC	
FvRAP-L4	gene10549	Forward	AGAATCCCTTTGGCCAGATT	196bp
		Reverse	CAGATTGCTGGGTGGTATTG	
FvRAP-L5/6	gene10550/1	Forward	AGCCGGTGAACATAAGAAGG	140bp
		Reverse	TTCCCTTTGAAGCGTACTCA	
FvRAP-L7	gene10552	Forward	GGATGTGTACGAGGCCCAGC	187bp
		Reverse	GGTCCATGAAGGGCGAGCAA	
FvRAP-RAP-L1	gene31672-28763	Forward	AGAGGGTGATGGTTTGCCTT	131bp
		Reverse	TTCGATTGCTGGAACTTGCC	
FvRAP-L1-RAP	gene28763-31672	Forward	GGCTCGAAGTCGAATCACAC	170bp
		Reverse	GGTGCTCTTGGACAGTCTCT	
Housekeeping	gene11892	Forward	AGCCTAACGCAGAGGTTCCAAA	136bp
		Reverse	GCAGCCCACATTGAAGGGTCTATAGT	
Actin2	At3G18780	Forward	TCCCTCAGCACATTCCAGCA	135bp
		Reverse	GATCCCATTCATAAAACCCCAGC	
